# Epithelial-to-Mesenchymal Transition in the Female Reproductive Tract: From Normal Functioning to Disease Pathology

**DOI:** 10.3389/fonc.2017.00145

**Published:** 2017-07-05

**Authors:** Olena Bilyk, Mackenzie Coatham, Michael Jewer, Lynne-Marie Postovit

**Affiliations:** ^1^Department of Oncology, University of Alberta, Edmonton, AB, Canada; ^2^Department of Obstetrics and Gynecology, University of Alberta, Edmonton, AB, Canada; ^3^Department of Anatomy and Cell Biology, Western University, London, ON, Canada

**Keywords:** epithelial-to-mesenchymal transition, adenomyosis, endometriosis, ovarian cancer, endometrial cancer, tumor microenvironment

## Abstract

Epithelial-to-mesenchymal transition (EMT) is a physiological process that is vital throughout the human lifespan. In addition to contributing to the development of various tissues within the growing embryo, EMT is also responsible for wound healing and tissue regeneration later in adulthood. In this review, we highlight the importance of EMT in the development and normal functioning of the female reproductive organs (the ovaries and the uterus) and describe how dysregulation of EMT can lead to pathological conditions, such as endometriosis, adenomyosis, and carcinogenesis. We also summarize the current literature relating to EMT in the context of ovarian and endometrial carcinomas, with a particular focus on how molecular mechanisms and the tumor microenvironment can govern cancer cell plasticity, therapy resistance, and metastasis.

## Introduction

In Western countries, the majority of malignancies affecting the female reproductive tract are comprised of ovarian and endometrial cancers (ECs). EC is considered to be the most commonly diagnosed cancer of the female genital tract with approximately 287,100 new cases worldwide (1, 2). Ovarian cancer (OC), on the other hand, with an estimated 140,200 deaths worldwide, is reported to be the most lethal gynecological carcinoma (2).

Both malignancies affect both premenopausal and postmenopausal women. A common feature of ovarian pathology in postmenopausal women is that it is frequently found with endometrial pathology, such as EC (3). Clinical studies have reported associations between hyperplasia of ovarian stroma with increased androgen production by the ovaries, which also coincides with the development of hormonally related tumors, such as EC (4). In 10% of all women with OC and 5% of women with EC, the cancers of the endometrium and ovary coexist (5). Pathological changes in the endometrium can sometimes occur as a consequence of OC. Ovarian and endometrial tumors do share common etiology related to reproductive factors such as number of ovulatory cycles, as well as hormone replacement therapy (6).

### Ovarian Cancer

In Canada, OC is only the eighth most commonly diagnosed cancer yet it ranks as the fifth leading cause of cancer-related death in women (7). Over 70% of women with OC are diagnosed with advanced-stage disease (FIGO stage III–IV) because of both a lack of symptoms and ineffectual screening at earlier disease stages (8). Despite recent advances in chemotherapeutical treatments using platinum-based drugs, taxanes, and targeted therapies, the 5-year survival rate for patients with OC is poor: 39–59% for stage III OC, and for stage IV OC, it is 17% (9). The aggressiveness of ovarian malignancy is associated with the development of chemoresistance along with tumor spreading into the peritoneal cavity encompassing both the pelvic and abdominal peritoneum. Transcoelomic and lymphatic spreading results in parenchymal liver/splenic metastases and extra-abdominal metastases, which are characterized as stage III and IV disease (10). Malignant ascites also occur frequently in OC patients (11, 12). Ascites act as a pro-inflammatory reservoir for countless soluble proteins, which makes ascites the perfect microenvironment to promote tumor cell metastasis (11, 12). Even though complete clinical remission is achieved in the majority of OC patients following initial therapy with paclitaxel plus carboplatin, over 75% relapse and develop progressive resistance to platinum-based chemotherapy leading to cancer dissemination, clinical relapse and eventually death (13).

Based on the dualistic model of ovarian carcinogenesis, ovarian tumors can develop either *via* a stepwise stochastic process from a borderline tumor to low-grade carcinoma (type I) or through a rapid *de novo* mechanism without defined precursor lesions (type II) (14). Type I tumors are made up of several different distinct histotypes, including low-grade serous, endometrioid, clear cell, mucinous, seromucinous carcinomas, and Brenner tumor. These tumors have good outcomes and are characterized by frequent mutations of the KRAS, BRAF, ERBB2, CTNNB1, PTEN, PIK3CA, and ARID1A genes, which trigger signaling cascades *via* the RAS/RAF/MEK/MAPK, PI3K/AKT, ARID1A, Wnt, PP2A and mismatch repair pathways. Notably, type 1 tumors lack *TP53* mutations (15–18). Type II tumors comprise high-grade (HG) serous carcinoma of the ovary, peritoneum, and fallopian tubes, undifferentiated carcinomas, and carcinosarcomas (15, 19). HG serous carcinoma is the most malignant type of epithelial ovarian carcinomas and accounts for up to 70% of all OCs (19). HG serous carcinomas are typically diagnosed at an advanced stage and are characterized by a high frequency of homologous recombination deficiency, TP53 mutations, activation of Notch3 and PI3K, and inactivation of RB and NF1 concomitant with tremendous genetic instability and intra-tumor heterogeneity. These features likely drive the poor outcomes associated with this disease subtype (20–22).

The dualistic theory of ovarian carcinogenesis proposes that serous OC is a heterogeneous disease arising from any of three potential sites: ovarian surface epithelium (OSE), fallopian tube epithelium, or mesothelium-lined peritoneal cavity (23). Emerging research suggests that endometrioid, clear cell, and seromucinous carcinomas are frequently associated with endometriosis with probable tubal origin, especially the lesions presenting as ovarian endometriotic cysts or endometriomas (18, 24).

Type II ovarian carcinomas account for most tubal and peritoneal cancers and seem to behave as one disease entity (25). In the peritoneum, metaplasia of presumed pluripotent stem cells has been linked to the promotion of synchronous malignant transformation at multiply foci, which in turn leads to peritoneal carcinomatosis (26).

Mechanisms governing the initiation and progression of OC are emerging in the extant literature. OC is a molecularly complex malignancy with phenotypic and functional heterogeneity arising among different histologic subtypes and among cancer cells within the same tumor (20, 27, 28). Intratumoral heterogeneity is a consequence of genetic mutations and reversible changes in cell properties, such as epithelial-to-mesenchymal transition (EMT), and alterations in extracellular matrix (29). Hypoxia and chemotherapy along with the elements of the tumor microenvironment (immune, perivascular or vascular cells, stroma, and extracellular matrix components) can drive EMT and the production of new types of cancer cells, some of which behave like stem cells and contribute to chemoresistance and disease recurrence (30, 31).

### Endometrial Cancer

Despite primarily afflicting women over the age of 45 and after the onset of menopause, EC is the most frequently diagnosed gynecological malignancy in Western countries. In Canada, in 2016, it is estimated that 1,050 of the 6,600 women diagnosed with EC, will die from this disease (7). Increased life expectancy and the rising incidence of obesity have both contributed to an increase in the prevalence of EC. Although the 5-year survival rate is high at 90% for FIGO Stage I and II EC, approximately 10–15% of patients will experience recurrent metastatic disease (32). Taken together with FIGO Stage III and IV EC, these recurrent non-uterine confined and advanced-stage cases of EC have median survival that has been reported to barely exceed 1 year (33).

As with ovarian carcinogenesis, endometrial carcinogenesis has been proposed to follow a dualistic model and ECs can be grouped into two types based on immunohistochemical and molecular features (34). Linked to obesity, estrogen excess and hormone receptor positivity, Type I endometriod ECs have more favorable outcomes than Type II serous tumors that are found mostly in older women (34). Treatment of early stages of Type I ECs has primarily been adjuvant radiotherapy whereas advanced stages of Type I and serous Type II tumors are frequently targeted by chemotherapy (35). In order to apply appropriate treatment to EC patients, proper subtype classification has been further supported by the characterization of commonly mutated genes within each histological subtype. Type I ECs frequently contain PTEN mutations coexisting with mutations to other genes in the P13K-Akt pathway (36, 37). Mutations to FGFR2, ARID1A, CTNNB1, PIK3CA, PIK3R1, and KRAS are also common in Type I tumors whereas TP53, PIK3CA, and PP2R1A mutations are most frequent in Type II ECs (38–42).

Further characterization at the molecular level using multiple platforms has provided an even more refined subdivision of ECs into different subtypes. Examination of somatic copy number alterations (SCNA) and microsatellite instability (MSI) resulted in EC clustering into four groups (35). One group consisted of mostly serous EC with extensive SCNA and low mutation rates (35). The remaining endometriod tumors could be divided into three subtypes: (1) ultramutated EC with very high rates of mutations; (2) a group of hypermutated MSI EC; and (3) microsatellite stable EC with low frequencies of mutations (35). Only the ultramutated subtype has progression-free survival near 100%, which strengthens the notion that a better understanding of the other subtypes is required to improve therapeutic application to patients who present with EC tumors genomically classified in this manner.

As the genomic contribution to aggressive forms of EC is being elucidated, a growing understanding of the other molecular and microenvironmental contributions to these tumors is also coming to light. Similar to OC, certain cases of EC display a great degree of heterogeneity at the phenotypic level. For example, undifferentiated endometrial carcinoma (UEC) exhibits a solid growth pattern lacking appreciable features of differentiation juxtaposed to well differentiated lesions (43). Evidence in the literature is building a strong case that the interplay between genetic mutations, aberrations to signaling factor activity and cues from the tumor microenvironment can drive EMT, or changes to the extracellular matrix of EC cells. The reported aggressive clinical behavior of UEC could be explained by EC having undergone EMT to become undifferentiated, motile stem-like cells that in turn do not respond to conventional chemotherapy.

## EMT in the Female Reproductive Tract

**Figure 1:**
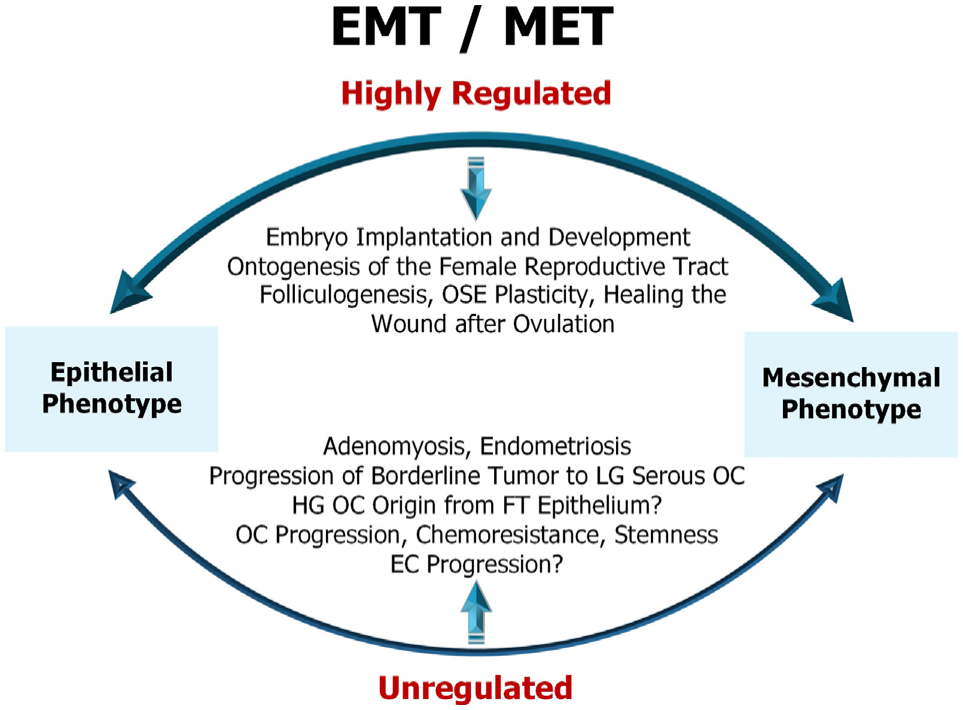
Representative diagram of epithelial-to-mesenchymal transition (EMT)/mesenchymal-to-epithelial transition (MET) in female reproductive tract. EMT/MET are highly regulated physiological processes in embryo implantation and early development as well as in the reproductive function of the ovary and endometrium. Dysfunction of EMT in the normal epithelial cells of the ovary and uterus results in pathological processes, such as adenomyosis, endometriosis, cancer initiation, progression, and resistance to therapy. OSE, ovarian surface epithelium; LG serous OC, low-grade serous ovarian cancer; HG OC, high-grade ovarian cancer; FT epithelium, fallopian tube epithelium; EC, endometrial cancer. For a detailed description of the diagram and references refer to Sections “Physiological (Non-Malignant) EMT in the Female Reproductive Tract” and “EMT in Cancers of the Female Reproductive Tract.”

As illustrated (Figure 1), EMT is a highly regulated physiological process in the early embryonic development and ontogenesis of the female reproductive tract (44). In adult organisms, EMT is important for folliculogenesis and occurs as a physiological response to injury during the wound healing after ovulation (44). Dysfunction of EMT in the normal epithelial cells of the reproductive organs, the ovary and the uterus results in pathological processes such as adenomyosis, endometriosis, cancer development, and metastasis (45). This will be discussed in detail further in the review.

During EMT, epithelial cells not only lose their polarity but also their adhesion to adjacent cells and the basement membrane and acquire properties that promote migration and invasion. These phenotypic changes are marked by the acquisition of a fibroblast-like mesenchymal appearance and cellular plasticity (45). In cancer cells, this developmental process is hijacked, allowing the tumor cells to dissociate, migrate, and metastasize (46, 47). Furthermore, EMT induces the emergence of cancer stem cell (CSC) traits, prevents apoptosis and senescence, induces resistance to chemotherapy, and contributes to immunosuppression (48).

**Figure 2:**
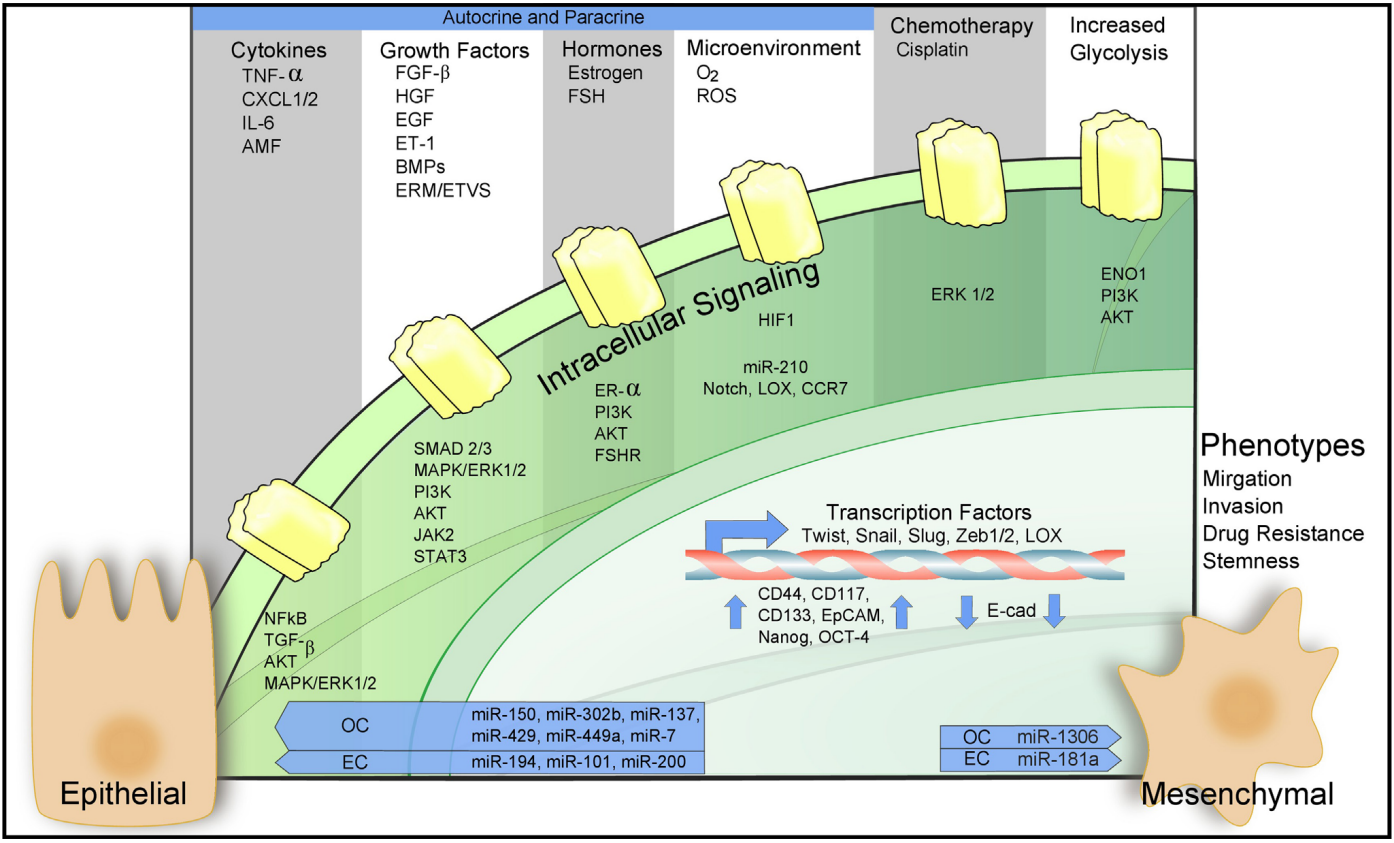
Epithelial-to-mesenchymal transition (EMT) in ovarian and endometrial cancers (ECs). Regulation of EMT-inducing signaling pathways through autocrine–paracrine signaling, chemotherapy, increased glycolysis, and the action of microRNAs. For a detailed description of the diagram and references refer to Sections “EMT in the Female Reproductive Tract,” “Molecular Mechanisms Governing EMT in Ovarian and ECs,” and “Microenvironmental Regulation of EMT in Cancers of the Female Reproductive Tract.”

Epithelial-to-mesenchymal transition is regulated epigenetically, transcriptionally, and post-transcriptionally. Downregulation of epithelial cell-specific tight and adherens junction proteins like E-cadherin in conjunction with the novel expression of mesenchymal proteins Vimentin and N-cadherin are trademark responses to the EMT program (49, 50). Numerous signaling pathways including PI3K/Akt, transforming growth factor β (TGF-β), EGF, hepatocyte growth factor (HGF), MAPK/ERK, NF-kβ, Wnt, Notch, estrogen-receptor-α (ER-α), and HIF-1α cross talk to participate in EMT upregulation. For a summary of these processes in the context of ovarian and ECs, see Figure 2. These signaling pathways act to mobilize embryonic transcription factors as well as epigenetic modifiers to reprogram epithelial cells toward a more mesenchymal-like fate (51).

## Physiological (Non-Malignant) EMT in the Female Reproductive Tract

### EMT in the Ovary

An overview of non-cancerous EMT and MET inducers in the female reproductive tract is presented in Table 1. The ovary consists of cells with different embryologic origins: OSE, stroma, germ cells, and sex cords (52). OSE is formed from celomic epithelium and ovarian stroma arises from the subcoelomic mesoderm. Invaginations of celomic epithelium in the superficial ovarian cortex form the sex cords (pregranulosa cells). In addition to being the progenitor of granulosa cells *via* the fetal OSE, the celomic epithelium, in the vicinity of the presumptive gonads, invaginates to give rise to the Mullerian ducts, which give rise to the oviduct, endometrium, and endocervix (52).

**Table 1:** Molecular and environmental inducers of non-cancerous epithelial-to-mesenchymal transition (EMT) and mesenchymal-to-epithelial transition (MET) in the female reproductive tract.

Inducers of EMT and MET	Targeted pathways	Function	Reference
**Ovary**			
transforming growth factor β (TGF-β) superfamily proteins: TGF-β1, Bone morphogenic proteins; Connexin43	SMAD; Tuberous sclerosis complex/mTORC1; ERK1/2	Granulosa cell growth and differentiation;follicular development	(61, 62, 67)
TGF-β, epithelial growth factor (EGF), collagen	Matrix metalloproteinase (MMP)-2,9; ERK1/2; Integrin-linked kinase	Ovarian surface epithelium motility, migration, proliferation and remodeling the extracellular matrix to heal the wound after ovulation	(63, 86, 88)
**Endometrium**			
Wnts, β-catenin	Wnt	Mullerian duct differentiation and uterine development	(74–78)
Steroid hormones (estrogen, progesterone)	MCL-1, STAT3	Embryo implantation	(83)
**Adenomyosis, endometriosis**			
β-catenin	Wnt	Endometrial gland hyperplasia	(98)
17β-estradiol	Estrogen-receptor-α	Migration and invasion of endometrial cells	(99)
LOXL1, LOXL4	TGF-β, To be discovered	Early stages of EMT	(106, 107)
Lipocalin2	MMP-9, To be discovered	Migration and invasion of endometrial cells	(109, 110)
Menstrual effluent, TGF-β (peritoneal endometriosis)	Src tyrosine kinase; SMAD; JNK1	Mesothelial cell motility	(112–116)
Oxidative stress (possibly involved in tubal origin of ovarian endometriosis)	ERK1/2	To be discovered	(121, 122)

Landmark animal model studies of EMT during fetal development and under physiological conditions have demonstrated that formation of primordial follicles is a multi-step process. Somatic cells originating from the surface of the ovary that surrounds the oocytes are recruited, where the OSE cells then subsequently undergo EMT and ingress into the stroma of the ovary (53, 54). During this process, two distinct pools of primordial follicles are formed: granulosa cells that populate the medullary pool of follicles are activated during the neonatal period, while granulosa cells in the cortical pool are activated after puberty (55–57). Fertility throughout the reproductive life of a female is accomplished once the primordial follicles at the ovarian cortex have been replaced by the first pool of follicles which then subsequently dominate the ovary following 3 months of age (55).

Several studies have revealed that granulosa cells undergo a partial EMT during folliculogenesis, and this transition is only completed with the rupture of the basal lamina and subsequent formation of a corpus luteum at the time of ovulation (57, 58). The study of Mora et al. (53) has demonstrated the distribution of epithelial and mesenchymal proteins during ovarian follicle development. Surprisingly, granulosa cells did not express epithelial markers E-cadherin, Cytokeratin-8, ZO-1, and expressed Vimentin suggesting a more mesenchymal phenotype.

Another study revealed that folliculogenesis is associated with two growth factors: epidermal growth factor (EGF) and keratinocyte growth factor (KGF) (59). EGF is produced by granulosa cells of primordial follicles helping precursor theca cells to be recruited to the follicle while KGF is a mesenchymal factor produced by theca cells promoting transition from primordial to primary follicle (59).

Recent findings elucidated the importance of TGF-β, a predominant stimulus of EMT, in regulating granulosa cell growth and differentiation, as well as the plasticity of OSE under physiological conditions (60–63). TGF-β is a cytokine whose many functions besides EMT include inducing growth arrest and fibrosis of tissue through complex canonical SMAD-mediated and non-canonical signaling pathways that cross talk with multiple growth factor-signaling pathways, including Wnt- and epidermal growth factor (EGF)-signaling pathways (64). TGF-β-SMAD signaling activates the expression of EMT transcription factors ZEB1, Snail, Slug, and Twist, which can repress the expression of microRNAs (miRNAs) that target mesenchymal components (65). Expression of particular sets of miRNAs that are capable of repressing the expression of epithelial proteins can also be triggered by TGF-β (65). Non-SMAD signaling pathways activated by TGF-β include PI3K-Akt-TOR complex 1 (mTORC1), MAP kinase and Rho-like GTPase signaling pathways (45, 66).

In the normal ovary, TGF-β and its receptors are expressed in human granulosa cells. Mouse models have shown that TGF-β maintains the dormant pool of primordial follicles through tuberous sclerosis complex/mTORC1 signaling in oocytes as well as downstream SMAD signaling pathways (60, 62). The most active TGF-β superfamily pathway in early human folliculogenesis consists of growth differentiation factor 9 combined with bone morphogenic proteins (BMP-15, BMP-4, and BMP-7), which all promote the intracellular activation of SMAD3 and SMAD4 (61, 67).

*In vitro* studies have reported that early OSE passages express *de novo* E-cadherin and also establish tight junctions exhibiting Claudin-1 and Occludin. Stimulation of OSE culture with TGF-β1 downregulates these epithelial markers and also prevents the formation of an epithelial barrier, leading to a mesenchymal phenotype in OSE cells. This phenomenon is driven by an EMT-like process and an altered molecular composition of the epithelial junction complex that partly coincides with the expression pattern of the naïve OSE (63). Co-expression of Cytokeratin-8 and Vimentin in naive OSE indicates a mixed epithelial–mesenchymal phenotype (68, 69) and it has been suggested that the OSE phenotype is highly plastic, switching between mesenchymal and epithelial states as a result of external factors such as TGF-β1. This TGF-β1-induced plasticity may also serve to drive OC progression (63).

### EMT in the Endometrium, Embryo Implantation, and Development

The mucosal endometrial lining of the uterus is highly regenerative due to the fact that it grows up to 7 mm in thickness in response to cyclical changes of plasma sex steroid hormone levels (70). It is a highly dynamic tissue, undergoing well-defined periods of proliferation, differentiation, and menstruation or shedding (71). Proliferation is influenced by the presence of estrogen during the follicular phase of the ovarian cycle, whereas progesterone stimulates differentiation during the secretory phase (72). Naturally, the cells comprising the endometrium have to retain a certain degree of plasticity at all times to be able to adapt to the cyclic hormonal changes. Accordingly, it has been hypothesized that mesenchymal-to-epithelial transition (MET) may play a major role in the compositional landscape of the endometrium, from its initial development to embryo implantation following the proliferative stage.

Mesenchymal-to-epithelial transition, a process in which mesenchymal cells become reprogramed to acquire epithelial traits, has been shown to be fundamental during embryo development (73). This cellular transdifferentiation process has also been confirmed to take place prenatally during development of the oviducts, uterus and anterior vagina from the Mullerian ducts (74, 75). The Mullerian ducts arise from the celomic epithelium, which originally developed from the intermediate mesoderm through MET (74, 75). EMT is then required to transform the celomic epithelium into Müllerian duct mesenchyme (MDM) (74, 75). Wnt signaling is known to be important for female reproductive tract development with Wnts expressed in both the Mullarian duct epithelium and mesenchyme. Wnt5a is essential for epithelial–mesenchymal paracrine interaction, while Wnt7a has a role in endometrial gland formation along with the patterning and morphogenesis of the female reproductive tract (76, 77). In mouse models, by stabilizing the levels of a downstream effector in the Wnt-signaling pathway, β-Catenin (CTNNB1), Wnt signaling was found to be critical for Mullerian duct differentiation and uterine development (78). In these models, it also appears that EMT is extended when CTNNB1 is stabilized in MDM. Hence, the normal development of the reproductive tract from the Mullerian duct is halted as the endometrial cells retain their mesoepithelial character (78).

Mesenchymal-to-epithelial transition has also been linked to embryo implantation in the uterus. If an embryo is to be implanted onto the endometrium a complex interplay between the endometrium and blastocyst must take place. *In vitro* implantation assays using human endometrial epithelial cells (EECs) have been designed to study early events in implantation such as the initial adhesion of the embryo (79). Through stimulation by steroid hormones and the histone deacetylase inhibitor, suberoylanilide hydroxamic acid, which promotes differentiation, an artificial system to study implantation has been constructed. Application of steroid hormones resulted in upregulation of Vimentin and N-cadherin, indicative of EMT, which allows the EECs to adjust their cellular polarity prior to receiving the embryo (79).

Decidualization is a critical process that must be undertaken at the site of implantation to allow for the pregnancy to progress naturally following the initial attachment phase. Contact between the blastocyst and the endometrium signals endometrial stromal cells to begin undergoing decidualization (80). In mouse models, MET prepares luminal epithelial cells for the change of shape and intercellular junctions that are necessary to adapt the uterus to support pregnancy (81). By transitioning to epithelial cells expressing high levels of Cytokeratin and low levels of Vimentin, these endometrial cells become more adherent, permitting the formation of coherent layers through intercellular adhesion complexes (82). Thus following MET, these endometrial cells are more susceptible to deeper penetration by the embryo. A hypothesis is emerging as to how the endometrial cells switch from a mesenchymal phenotype to becoming primarily epithelial in nature. A direct interaction between the transcription factor, STAT3 and MCL-1, a gene discovered to be expressed in cells committed to differentiation, has been proposed to be responsible. This is due to the fact that in the presence of the steroid hormones, estrogen and progesterone, MCL-1 and STAT3 can co-localize to the nucleus and modulate the promoter activity of STAT3 (83). Overexpression of the two proteins prevents EMT and leads to an increase in epithelial markers along with a concurrent downregulation of mesenchymal markers (83). As co-localization of MCL-1 and STAT3 is most evident in stromal cells post-implantation, during decidualization, these two factors have been implicated in the EMT to MET shift necessary for successful embryo implantation (83).

Interestingly, the ability of endometrial cells to exist in both epithelial and mesenchymal phenotypes by undergoing timely switches between EMT and MET, allows the endometrium as a whole to acquire the cellular traits necessary to develop healthy gland architecture and successfully accept embryos for implantation.

### EMT in Ovulation and Menstruation and Its Dysregulation in Adenomyosis and Endometriosis

Following monthly ovulation, ovarian surface epithelial cells surrounding the newly erupted follicle undergo EMT. This EMT event is induced by the local microenvironment and allows the cells to manifest a phenotype, which resembles fibroblasts and fosters motility, migration, proliferation and the ability to remodel the extracellular matrix to heal the wound after ovulation (84). Through ischemia-reperfusion associated with ovulation-induced injury, a complex interplay of proteolytic enzymes and inflammatory molecules, such as bradykinin, prostaglandins, and leukotrienes are generated during ovulation (85, 86).

Transforming growth factor-β, leukemia inhibitory factor (LIF), EGF and extracellular matrix components such as collagen play key roles at the site of ovulatory rupture, inducing EMT in OSE (63, 87, 88). Gamwell et al. (87) identified a progenitor cell-like population of mouse surface epithelial cells that expresses stem cell marker lymphocyte antigen 6 complex, locus A (LY6A) and might be responsible for ovulatory wound healing. TGF-β and LIF, two factors in the follicular fluid have been demonstrated to modulate the size of the LY6A expressing progenitor cell population (63, 86).

EGF in conjunction with hydrocortisone is a major EMT-inducing factor and causes the acquisition of a fibroblast-like morphology. Moreover, EGF increases cell motility and enhances the activity of secreted pro-matrix metalloproteinase (MMP)-2 and -9 while also augmenting activation of ERK and integrin-linked kinase (88).

Despite the fact that the endometrium is one of the female body’s most dynamic organs, very little is known with regard to how EECs regenerate themselves during the menstrual/estrous cycle. As menstruation results in the loss of the upper two thirds of the endometrium, this region of the uterus is a constant site of physiological injury and thus requires repair (89). Re-epithelialization has to occur quickly to repair the endometrium and appears to act independently of estrogen hormone levels (90). Within the endometrium, epithelial cells line glands and are further supported by a substantial network of vascularized stroma (91). MET is a common process during uterine development and embryo implantation; hence it is not surprising that it also occurs during the menstrual cycle. This was demonstrated through fate-mapping where MDM-derived cells near the stromal-myometrial border were found within the glandular and luminal epithelium following endometrial regeneration (92). This phenomenon of stromal cells adjacent to the epithelium transitioning from a mesenchymal-to-epithelial state has also been observed in murine models (93). The role that the shedding layer plays in the stimulation of MET and repair has remained elusive, and should be studied to gain greater insight into this process.

With emerging evidence demonstrating that MET, and to some extent EMT, regulates endometrial composition and regeneration under normal, dynamic uterine conditions, such as embryo implantation and the menstrual cycle, it is possible that dysregulation of signaling pathways that induce EMT could result in such uterine pathologies as adenomyosis, endometriosis, and eventually EC. A common and benign gynecological disorder dependent on estrogen levels, adenomyosis, occurs when the normal uterine boundaries are disrupted and endometrial glands and stroma are found within the myometrium (94, 95). This affliction is usually associated with difficulties in implantation and therefore leads to reduced fertility (96).

It is likely that aberrant EMT events play a role in the evolution of adenomyosis. As previously mentioned in the context of uterine development, elevated levels of β-catenin (CTNNB1) can result in the activation of the Wnt pathway, which can ultimately lead to aberrant activation of EMT (97). One of the first steps toward an EMT event has been hypothesized to be the translocation of β-catenin to the nucleus as part of the Wnt-signaling pathway and its subsequent accumulation (97). This would disturb the balance of β-catenin that normally interacts with E-cadherin in cell–cell adherens junctions. β-catenin activation and induction of EMT have been proposed to play a major role in the pathogenesis of adenomyosis. Conditionally stabilizing levels of β-catenin in mice has led to both endometrial gland hyperplasia and infertility being observed along with decreased expression of E-cadherin and induction of Snail and ZEB1, which further repress E-cadherin levels in EEC (98). EMT can also be induced by estrogen through upregulation of the transcription factors, Snail or Slug (99). In human tissue samples, markers of EMT, E-cadherin loss in particular, emerged in response to 17β-estradiol and selective estrogen receptor modulator abolished EMT, migration and invasion of ER-positive endometrial cells (99). Thus, above average levels of steroid hormones could be a predicative factor of adenomyosis and the focus of research on possible therapies.

Presently in endometriosis, lysyl oxidase (LOX) isoforms and Lipocalin2 have been tenuously linked to EMT, which would allow the epithelial cells to gain the necessary migratory and invasiveness properties to take root outside of the uterine cavity (100, 101). It has been confirmed by RT-PCR and IHC that in human samples of endometriosis, N-cadherin along with the transcription factors, Twist, Snail and Slug are all upregulated when compared to healthy endometrial epithelial and stromal cells (100). This result is independent of the status of E-cadherin levels suggesting that downregulation of E-cadherin leading to EMT is not a general feature in the development of endometriosis (100). Depending on the microenvironment of endometriotic lesions, it would seems that EMT arises in response to stress and leads to EECs becoming invasive and gaining metastatic competency (101). The five isoforms of LOX family proteins have been shown to cooperate with Snail in repression of E-cadherin expression (102, 103). Two isoforms in particular, LOXL1 and LOXL4, have been linked to the endometrium, either becoming downregulated during implantation or possessing single-nucleotide polymorphisms associated with the onset of endometriosis (104–106). Overexpression of LOX in endometrial cell lines did not result in the full activation of EMT with E-cadherin only weakly downregulated (107). It is now being hypothesized that LOX can only bring about the early stages of EMT and other factors are required to fully induce extracellular remodeling of the endometrium (107). Lipocalin2 was discovered to be upregulated in endometriotic lesions from gene profiling experiments performed in rat models of endometriosis (108). This protein is thought to be a stress factor affecting cellular physiology in response to environmental changes (109). *In vitro*, nutrient deprivation stress induces EMT in EC, which in turn can lead to endometriosis, if the immune system is hijacked and high levels of anti-apoptotic cytokines allow for abnormal growth of endometrial tissue outside of the uterus (110). Lipocalin2-mediated triggering of EMT likely contributes to the implantation of the ectopic endometrial tissues by inducing migration and invasion properties in ECs. Changes in Lipocalin2 expression directly correlate with EMT markers in stressed EECs supporting the notion that Lipocalin2 can induce EMT to generate the morphological and physiological changes in ECs required to drive endometriosis (110). Presently, Periostin, a secretory extracellular matrix protein found at high levels in ectopic endometrium of endometriosis, has been linked to induction of EMT in endometrial stromal cells through integrin and Akt pathways (111). The role of Periostin in the facilitation of EMT in endometrial epithelial is still up for debate (111).

*In vitro*, it has been demonstrated that menstrual effluent can promote EMT of peritoneal mesothelial cells (MC) increasing MC motility, changing the distribution of cytokeratins, fibrillar actin and α-tubulin and upregulating Snail and Vimentin expression (112, 113). It has been postulated that an increased exposure to a retrograde flow of menstrual effluent may lead to an insult for the mesothelium. Accordingly, there is an increased risk for pelvic endometriosis when menstrual periods are longer and blood flow is heavier (113).

In peritoneal MC in animal models, a cross talk between JNK1 and SMAD3 pathways during TGF-β1-induced EMT has been demonstrated (114). The role of TGF-β in the etiology of peritoneal endometriosis has also been demonstrated in women affected by endometriosis (115). TGF-β1 level is high in the peritoneal fluid of women with endometriosis and *in vitro* peritoneal MC also secrete TGF-β1. Moreover, the TGF-β-stimulated SMAD2/3 signaling pathway is active in the peritoneum and genes associated with tumorigenesis (MAPK8, CDC6), EMT (Notch1), angiogenesis (ID1, ID3) and neurogenesis (CREB1) are all found to be upregulated in the peritoneum of women affected with endometriosis (115).

The impact of ovulation and menstruation on tubal epithelium has been assessed *in vitro* and with three-dimensional organ culture systems in animal models (116). This was the first study (116) that showed that ovulation is associated with inflammation that leads to DNA damage and genomic instability in the fimbriated end of the fallopian tube. It could be hypothesized that it occurs due to close proximity of the ovary to the fallopian tube epithelium, which is exposed to iron-induced oxidative stress generated from hemolysis of erythrocytes of menstrual blood by pelvic macrophages (117). *In vitro* treatment with hydrogen peroxide or macrophage-conditioned medium resulted in an increase in DNA damage in tubal epithelial cells (116). TP53 mutations are frequently found in precursor lesions of fallopian tube epithelium suggesting that they are an initiating events in serous tumorigenesis (118). Live-cell microscopy assays confirmed this hypothesis showing that expression of mutant TP53 in immortalized human fallopian tube epithelial cells promotes cell–cell aggregation and survival under cell detachment conditions. Subsequent mesothelial intercalation capacity was most likely occurring through a mechanism involving mesenchymal transition and matrix production (119).

As has been defined by Hanahan and Weinberg, oxidative stress is one of the hallmarks of cancer (120). Reactive oxygen species (ROS) can enhance proliferative qualities of cancer cells by transactivation of receptor tyrosine kinase and activation of ERK. As well, ROS have been shown to promote cell dissemination due to metalloproteinase secretion/activation and induction of EMT (121). The role of oxidative stress in malignant transformation through the induction of EMT and acquisition of stem cell characteristics has been demonstrated in human renal epithelial cells. Oxidative stress induces EMT in kidney epithelial cells concomitant with morphological changes and the upregulation of EMT-related transcripts (122). The role of ovulation-induced oxidative stress in the induction of EMT in fallopian tube epithelial cells requires further investigation as knowledge would lend mechanistic insight to the tubal origins of ovarian endometriosis and HG ovarian carcinoma.

In summary, EMT plays a key role in remodeling the extracellular matrix to repair OSE after ovulation, and key regulators of EMT in this process are TGF-β, EGF, and collagen. In contrast, in the endometrium, MET is a key regulator of regeneration following menstruation and a better understanding of the molecular players involved in this process is required. Activation of EMT by the dysregulation of the Wnt-signaling pathway has been implicated in the pathogenesis of adenomyosis, while LOX isoforms and Lipocalin 2 are the main inductors of EMT that have been implicated in the pathogenesis of endometriosis at this time. EMT of peritoneal MC has been shown to be activated by the Src tyrosine kinase and TGF-β signaling pathways and EMT could be involved in the pathogenesis of peritoneal endometriosis. In the distal end of fallopian tube, EMT induced by oxidative damage generated from hemolysis of erythrocytes in menstrual blood can possibly be linked to the onset of ovarian endometriosis with tubal origin and HG ovarian carcinoma.

## EMT in Cancers of the Female Reproductive Tract

### EMT in the Progression of Serous Borderline Ovarian Tumors to Low-Grade Serous Ovarian Carcinomas

Serous borderline tumors or low malignant potential tumors histologically are defined by atypical epithelial proliferation without stromal invasion. These cancers tend to be diagnosed at earlier stages and are characterized clinically by good prognosis and superior overall patient survival (123). Nevertheless, pelvic and abdominal recurrence may occur 10–15 years after the initial diagnosis and some patients eventually die from the disease (124). Epidemiologic and molecular data support that borderline tumors may give rise to invasive low-grade serous carcinoma. EGF and TGF-β have been shown to support this conversion, at least in part by promoting EMT and invasiveness in serous borderline ovarian tumor cells (125, 126). *In vitro* studies have demonstrated that migration and invasion of SBOT cells can be induced by EGF and TGF-β which promote EMT through activation of SMAD3, ERK1/2, and PI3K/Akt pathways. Activation of these signaling cascades leads to downregulation of E-cadherin and upregulation of transcription factors such as Snail, Slug, Twist, and ZEB1 (125, 126).

### EMT in HG Serous Ovarian Cancer

As described above, OC, in particular HG serous disease, is characterized by therapy resistance and metastatic progression. Tumor metastasis primarily occurs due to exfoliation of single malignant cells or cell clusters from the primary tumor into the peritoneal cavity. Cancer cells can subsequently attach to visceral and parietal peritoneal surfaces of the abdominal organs (127). Recent evidence suggests that peritoneal dissemination may also occur simultaneously, from tubal intraepithelial carcinoma, and often precedes ovarian carcinomas in HG OC (128).

In order for metastasis to occur, cancer cells must be able to locally invade out of the primary site. Importantly, they must also overcome anoikis (programmed cell death when cells detach from extracellular matrix) triggered within the ascites fluid in the peritoneal cavity. Finally, the cells must be able to attach to surfaces, such as the omentum (129). Recent studies by Pradeep and colleagues have demonstrated that OC metastasis can also occur *via* a hematogeneous route, wherein intravasation followed by cancer cell transit into blood vessels and extravasation into a secondary site occurs (130).

Both mechanisms of OC metastasis are dependent on motility and invasion involving EMT (127). EMT is considered to be the first step of the invasion cascade. In this process, primary tumor cells lose their cell-cell adhesions and able to migrate and invade the basement membrane. Once intravasated, these cells stay in the bloodstream as circulating tumor cells (CTCs). Micrometastases then occur in distant organs when CTCs exit the bloodstream and regain their epithelial characteristics (127).

Alternatively, the cells would use EMT in order to invade secondary sites in the peritoneum following attachment. Therefore, EMT and MET enable epithelial carcinoma cells to invade, disseminate, and colonize distant organs.

### EMT in EC

It can logically be hypothesized that the extreme invasiveness and poorer patient prognosis associated with HG EC and UEC is a direct result of EC cells having undergone the process of EMT. Although EMT can lead EC cells to lose their cell–cell adhesions and acquire the ability to migrate and proliferate, studies have not yet proven that EMT in EC cells will eventually result in metastases. More experimentation is required to gain a clearer picture of the role of EMT in EC cancer progression.

## Molecular Mechanisms Governing EMT in Ovarian and ECs

Transcription factors are among the best-characterized mediators of EMT (49). For example, Twist1, Snail, Slug, ZEB1, and ZEB2 all have been shown to repress the activity of E-cadherin leading to EMT (131). In OC and EC tissue specifically, overexpression of Twist, Slug, ZEB1, ZEB2, and Snail is linked to reduced expression of E-cadherin (132, 133). Nuclear β-catenin has also been shown to promote EMT by upregulating Slug expression (51, 134–137). SALL4, an essential transcription factor with a well-described role in the maintenance of pluripotent embryonic stem cells is aberrantly expressed in EC, promoting invasiveness through the up regulation of mesenchymal cell markers such as N-cadherin (138). SALL4 induces EMT through c-Myc, another transcription factor and oncogene (138).

Kruppel-like factor (KLF17) was initially thought to be an inhibitor of EMT and a tumor suppressor in several cancers including breast cancer (139–141). In the context of EC, KLF17 functions as a driver of EMT (142). EC tissue has elevated levels of KLF17 and expression of KLF17 in EC cell lines, leads to an upregulation of EMT-inducing transcription factors (142). Interesting, in OC cells, KLF4, a transcriptional factor related to KLF17, reduces cell proliferation, migration and invasion by attenuating TGF-β induced EMT (143). Hence, some transcriptional programs may regulate EMT in a tissue specific manner.

Another molecular player that has been shown to drive EMT is the neurotrophic receptor tyrosine kinase B (TrkB). Long recognized as an important oncogenic factor in a neurogenic context, when the TrkB signaling pathway involving the neurotrophic factor BDNF is activated in other tumor types, tumor cell proliferation, invasion, and metastatic potential are all stimulated (144). This pathway has also been linked to anoikis resistance in multiple cancers by inhibiting cell death and, therefore, leading to metastatic spread of cancer (145). TrkB and its high affinity ligand, BNDF are detected at high levels in both EC and OC (144, 146). TrkB levels determine the fate of EC cell lines, causing the Cadherin switch most commonly associated with an EMT event (144). The Akt and MAPK pathways are downstream of the TrkB signaling pathway, which could provide an explanation as to how the actions of several transcription factors can converge on the single yet complex cellular process of EMT.

Epithelial-to-Mesenchymal transition may also be regulated by metabolic processes. Enolase (ENO1), an enzyme functioning in the glycolytic pathway was hypothesized to have some role in tumor development. This idea was based on the logical observation that increased glucose uptake and aerobic glycolysis are characteristic features of rapidly growing cells (147). In EC, silencing ENO1 decreases Snail and N-cadherin expression while upregulating E-cadherin levels (148). At the same time, silencing ENO1 downregulates levels of proteins in the PI3K/Akt pathway, also resulting in Snail being expressed at lower levels (148). It is hypothesized then that ENO1 could be a potential oncogene, activating the PI3K/Akt pathway and eventually initiating downstream EMT signaling cascades in EC while in OC cells its role remains to be investigated.

Most recently, epigenetic modifications such as DNA methylation and the effects of non-coding RNA have been shown to be critical to the development of cancer (149). MiRNAs in particular have been found to be upregulated in many cancers, including both EC and OC, acting as oncogenes or tumor suppressor genes (150, 151). MiRNAs act as regulators, binding the 3′UTR region of coding RNAs triggering either the repression of mRNA translation or the degradation of the RNA completely (152). In general, high levels of miRNAs are associated with a variety of cancers but an understanding of how specific miRNAs regulate the expression of different oncogenes in EC and OC is gradually being uncovered.

Presently, EMT is suppressed in EC through the action of four miRNAs: miR-194, miR-101, miR-23a, and miR-124. miR-194 has been linked to B lymphoma mouse Moloney leukemia virus insertion region 1 (BMI-1), a protein associated with self-renewal and malignant transformation. In EC cell lines, BMI-1 can be linked to enhanced invasiveness, and miR-194 levels in highly invasive EC *in vitro* are inversely correlated with BMI-1 expression (153). miR-194 transfection decreases cell invasion in the HEC50B cell line while simultaneously inducing a loss of the EC cell line’s mesenchymal phenotype (153). miR-101 is downregulated in both endometriod and serous EC and has been found to inhibit proliferation of EC cells in the aggressive serous type. Notably, increasing miR-101 levels in EC cells reverses EMT (154). Specific to EC, miR-101 suppression of EMT can partly be linked to enhanced expression of EZH2, a histone-lysine *N*-methyltransferase enzyme that participates in histone methylation and, ultimately, transcriptional repression. EZH2 downregulates mesenchymal markers and Wnt/β-catenin signaling, leading to MET (154). miR-23a has also been found at significantly reduced levels in EC tissue (155). Overexpression of miR-23a *in vitro*, led to inhibition of EMT in HEC 1A cells through the targeting of SMAD3 (155). Downregulated in EC, miR-124 expression is partially attenuated by DNA methylation (156). Much like miR-23a, miR-124 when expressed at higher levels reverses the EMT-like phenotype, exhibiting reduced migration, invasion and proliferation through the upregulation of the scaffolding protein IQGAP1 (156).

Undifferentiated endometrial carcinoma frequently possesses a reduction in E-cadherin expression (157). MiRNAs have been implicated in the modulation of the epithelial differentiation status by repressing the action of ZEB1 and ZEB2, which are transcriptional repressors of E-cadherin (157). In particular, members of the miR-200 family inhibit the expression of ZEB1 and ZEB2. These transcription factors inhibit E-cadherin expression and thus drive EMT. Hence, miRNA-200 family members lead to a reduction and/or reversal of these processes (158). Of note, ZEB1 and ZEB2 can also bind to promoter regions of miR-200, leading to reduced expression. DICER1, a cytoplasmic RNase III enzyme responsible for cleaving miRNA into active 22 nucleotide species is also downregulated in undifferentiated EC (159). By preventing miR-200 processing, dysregulation of DICER1 leads to reduced E-cadherin levels concomitant with the upregulation of Vimentin, N-cadherin, Twist1, Snail and ZEB2. It should be noted that not all miRNAs inhibit EMT. For example, miR-130b, an oncogenic miRNA implicated in many advanced carcinomas, has been shown to drive EMT in EC by impairing E-cadherin expression (160). Therefore in EC, miRNAs’ regulation of oncogene expression can influence the induction of EMT and the ability of endometrial cells to acquire phenotypes with the potential to metastasize.

Various miRNAs such as miR-200 family and the miR-199/214 cluster regulate EMT in ovarian tumors (161). Several miRNAs have been reported to function as tumor suppressors, directly targeting EMT transcription factors thereby inhibiting cell invasion and metastasis (162). For example, miR-150 in OC cells can directly suppress ZEB1 (162). Other important tumor suppressor miRNAs are miR-302b and miR-137. Ectopic expression of these miRNAs in OC cells inhibits cell invasion, proliferation, and colony formation. They also promote apoptosis by targeting RUNX1 and modulating the activity of the STAT3 signaling pathway (163, 164). Some miRNAs are able to reverse the EMT process by targeting the Notch and Wnt-signaling pathways as has been demonstrated for miR-429 and miR-449a. MET, which is induced by these miRNAs, significantly increases drug sensitivity in metastasizing OC cells (165, 166). miR-7 has been shown to inhibit metastasis and reverse EMT through Akt and ERK1/2 pathway inactivation by reducing EGFR expression in OC cell lines (167).

Numerous miRNA profiling studies in HG serous OC have identified miRNAs associated with EMT induction, chemotherapy resistance, and disease progression. For example, miR-181a was identified as an inducer of EMT as it represses SMAD7, an inhibitor of TGF-β signaling (168). Ectopic expression of miR-181a increases cellular survival, migration, invasion and drug resistance. Therefore in OC, miRNAs can influence both inhibition and induction of EMT and are implicated in OC progression.

## Microenvironmental Regulation of EMT in Cancers of the Female Reproductive Tract

Induction of EMT results from a complex interplay between biophysical parameters such as hormones and hypoxia, biological agents such as tumor-infiltrating immune cells, and therapeutic interventions including chemotherapy (see Figure 2). The roles that these factors play in EMT will be discussed below.

### Hormones As a Driver of EMT in Ovarian and ECs

Experimental and clinical studies have revealed that cancer cells of hormone-sensitive tumors in the ovary, endometrium and breast hijack ER-α and β (ER-β) dependent pathways to promote proliferation, DNA repair, and cell survival (169–171). Moreover, the biologically most active estrogen (17 β-estradiol—E2) has been shown to stimulate EMT.

Estrogen-sensitive tissues are highly responsive to estrogen exposure, which can occur naturally during the menstrual cycle and pregnancy or as a result of obesity or the use of postmenopausal hormone replacement therapy (171). Studies of estrogen metabolism in postmenopausal women, has revealed that E2 can also be produced intracellularly by cancer cells using aromatase (172). In this pathway, circulating inactive plasma estrogen precursor E1S (estrone sulfatase) wchich is originated from peripheral tissues (liver, muscle, skin, bones) is converted to active E2 (172).

*In vitro* studies have demonstrated that E2 exposure drives EMT in the OC cell line BG-1. Upon E2 stimulation, a decrease of E-cadherin expression was observed in conjunction with a significant upregulation of the EMT-associated transcription factors Snail and Slug (173). These changes lead to a mesenchymal phenotype as well as increased invasiveness. More importantly, ER-α activation has been shown to promote EMT as well as stem cell traits in OC cells (173). Specifically, knockdown of ER-α in OC cells decreased N-cadherin expression, and increased E-cadherin expression (174). Furthermore, knockdown of ER-α significantly reduced the formation of CSC-enriched spheres and decreased expression of Nanog, BMI-1, and Oct-4 stem cell markers. Follicle-stimulating hormone (FSH) can also induce EMT-like phenotypes in OC cells by activating the PI3K/Akt-Snail signaling pathway (175). FSH receptor is present in the majority of OCs, and FSH is an important ovarian epithelial growth-promoting factor (176). In opposition to E2 and FSH, progesterone has been shown to inhibit EMT in OC cells *via* a progesterone receptor-dependent pathway (177). It has been demonstrated that Vimentin expression is reduced upon treatment with progesterone, while E-cadherin expression is increased.

Estrogen and progestins can also upregulate ZEB1 in the stroma and myometrium of the uterus and in human cells *in vitro* (178, 179). Interestingly though, there are no hormone response elements upstream of the translational start site of ZEB1. In aggressive cases of EC, such as grade 3 endometrioid and type II serous carcinomas, ZEB1 overexpression is not limited to the stroma and myometrium. Indeed, the ZEB1 protein is aberrantly expressed in epithelial-derived carcinoma cells as well (178). Loss of E-cadherin expression paired with ZEB1 expression in a high percentage of epithelial cells is characteristic of EMT and suggests hormonal regulation of the entire process.

During the normal menstrual cycle, the steroid hormone, progesterone can induce differentiation in EC cells. Progesterone induces the expression of inhibitors of Wnt signaling which in turn downregulate EMT and slow down cancer progression (180). Loss of progesterone receptors has been found in patient tissues with progressive EC and also witnessed in these cases, is a significant upregulation of pathways involved in the progression of cells to a mesenchymal phenotype (180). Taken together with the fact that application of medroxyprogesterone acetate, a synthetic variant of progesterone, to EC cells *in vitro* inhibits migration and downregulates Vimentin; a strong case for progesterone-mediated inhibition of EMT can be presented (180). Progesterone also downregulates TGF-β, a signaling pathway which is a major driving force behind EMT.

Much like in OC, elevated levels of E2 contribute to the enhanced proliferation and invasive capasity of EC cells through the activation of the PI3K/Akt and MAPK signaling pathways and mass and obesity-associated (FTO) gene expression (181). A study by Wik and colleagues (181) not only provided new insight into the mechanisms of E2-induced proliferation and invasion of EC but also provided a link to obesity. However, unlike OC, lack of ER-α in EC correlates with activation of Wnt-, Sonic Hedgehog- and TGF-β signaling pathways and induction of EMT suggesting ER-α independent mechanisms of EMT regulation (181). Most recently, conditioned media from normal endometrial stromal factors has been found to inhibit estrogen-induced EMT through regulation of Slug and E-cadherin expression levels (182). Metformin, a commonly used drug used to treat type 2 diabetes can also reduce E2-induced cell proliferation and EMT in EC cells through suppression of ERK1/2 signaling and activation of AMPKα signaling (183).

Collectively, these studies provide a compelling argument for the role of estrogen in promoting tumor progression by induction of EMT and in particular highlight the critical role of ER-α in OC and EC progression.

### Cytokines/Chemokines and Growth Factors As Drivers of EMT in Ovarian and ECs

Inflammatory cytokines are low-molecular weight proteins produced by both immune (tumor-infiltrating T-regulatory lymphocytes and activated macrophages) and stromal cells [cancer-associated fibroblasts (CAFs) and vascular endothelial cells] in the tumor microenvironment (184). Along with a wide variety of tumor cells, cytokines contribute to proliferation, cell survival, differentiation, immune cell activation, and cell migration, thus supporting tumor growth, progression, and adaptation (185).

The human OC microenvironment contains a dynamic inflammatory cytokine network that has been described in recent studies as the “TNF network.” In this network, key cytokine/chemokine mediators of cancer-related inflammation such as interleukin 6 (IL-6), tumor necrosis factor-α (TNF-α) and stromal cell-derived factor-1 (CXCL12) promote disease progression by facilitating bidirectional communication between the tumor and the stroma (185).

Tumor necrosis factor-α is a major inflammatory cytokine that promotes cancer cell migration and invasion. Prolonged production of TNF-α in the tumor microenvironment results in increased myeloid cell recruitment in an IL-17-dependent manner (186). Moreover, a high level of TNF-α in combination with IL-6 in ascites’ fluid and tumor tissue of OC patients is associated with tumor progression and resistance to chemotherapy concomitant with shortened progression-free survival (187, 188). TNF-α facilitates tumor cell migration and invasion by inducing the transcriptional upregulation of genes associated with EMT such as Snail, Twist, ZEB1, and ZEB2. This upregulation is dependent upon activation of the NFκB and TGF-β signaling pathways (189, 190). A recent study in colorectal carcinoma demonstrated that TNF-α increases stability of Snail through the activation of Akt pathway and repression of GSK-3β activity (191). Furthermore, exposure to TNF-α stimulates the MAPK/ERK signaling pathway, which results in a positive feedback loop that helps drive EMT in colon carcinoma spheroids (192). Similarly, in OC, autocrine production of TNF-α within a tumor stimulates a constitutive network of cytokines, chemokines, and angiogenic factors in the stroma. These factors act to promote EMT, colonization of the peritoneum, and neovascularization of metastatic lesions (193). The chemokine CXCL12 has also been shown to promote EMT, cell proliferation, migration, and invasion of OC cells (194–196). These effects of CXCL12 occur *via* a MAPK/ERK-dependent pathway.

As was outlined in Ref. (197), growth factors such as TGF-β, epidermal growth factor (EGF), HGF, and endothelin-1 (ET-1) are all important inducers of EMT in OC. Of these, members of the TGF-β superfamily have been studied extensively. By regulating cell growth and death and repressing the expression of oncogenes, TGF-β acts a tumor suppressor in normal cells and in the early stages of carcinogenesis. As the tumor develops and progresses, these protective effects of TFG-β are often lost leading to resistance to the TGF-β growth inhibitory effect concomitant with TGF-β-mediated promotion of cell migration, invasion, and metastasis (198).

By Western blot analysis, TGF-β protein has been identified not only in the cell lysates obtained from OC cell lines but also in their culture media, suggesting that TGF-β is produced by OC cells (199). TGF-β contributes to ovarian tumor growth through a number of mechanisms. For example, it is a potent activator of CAFs (200). CAFs isolated from OC tissues have been shown to induce cancer cell invasion and migration while fibroblasts isolated from normal ovarian tissue did not show this ability *in vitro* (201). Yeung et al. have used coculture experiments to demonstrate that in the tumor microenvironment TGF-β facilitates a cross talk between OC cells and CAFs, promoting the motility and invasion of OC cells by upregulating Versican in CAFs (200).

Several studies suggest that TGF-β drives dissemination of OC. For example, microarray analyses (GSE2109) revealed that TGF-β signaling pathway is activated after dissemination of cancer cells from primary site into the peritoneal cavity (202). Expression of TGF-β receptor type 2 and phosphorylated SMAD2/3 were upregulated in omental metastases, suggesting a role for autocrine signaling at metastatic sites. Another study demonstrated that overexpression of the homeodomain transcriptional factor, PITX2 results in the gain of mesenchymal phenotype in OC cells, leading to increased cellular invasion. These effects of PITX2 are due to the activation of the TGF-β pathway (203).

Several mechanisms have been shown to mediate the pro-tumorigenic effects of TGF-β in OC. Treatment of OC cell lines with TGF-β and EGF upregulates the gap junction protein Connexin43 (Cx43) by activating SMAD2/3, ERK1/2, and Akt signaling pathways. This upregulation in Cx43 then promotes cancer cell migration and proliferation (204, 205). Epigenetic mechanisms have also been shown to underline TGF-β-induced EMT as alterations in the expression of genes associated with EMT induced by global and gene-specific DNA-metylation (200, 206).

Additional TGF-β family proteins might also regulate EMT in OC cells. For example, increased production of BMPs by OC cells and surrounding stroma has been shown to promote tumor growth (207). Investigating the effect of BMP-2 on OC tumorigenesis *in vitro*, Le Page et al. (208) demonstrated that treatment of OC cells with recombinant BMP-2 induces phosphorylation of SMAD1/5/8 and ERK/MAPKs results in the upregulation of Snail concomitant with increased cellular motility. BMP-4 has been shown to alter the morphology of OC cells by inducing EMT markers such as Snail and Slug and promoting an invasive phenotype. Furthermore, the BMP-4 inhibitor, Noggin, blocks BMP-4-induced EMT phenotypes, and decreases autocrine BMP-4-mediated OC cell motility (197, 209).

Nodal, is another member of TGF-β superfamily, and a morphogen during early embryonic development (210). Nodal promotes cellular invasion and EMT concomitant with the induction of Snail, Slug, and Twist in several cancers including breast cancer and melanoma (210–212). Nodal overexpression in OC cells has been associated with decreased proliferation (213); however, studies have not yet examined the role of this morphogen in OC EMT. In EC, Nodal expression is positively correlated with epidermal growth factor-co-receptor Cripto and increases dramatically in the transition from histologic Grade 1 through 4 (214).

Several other growth factors have been shown to work independently or in combination with TGF-β to induce EMT in OC. EGF promotes EMT and cancer cell migration directly by activating EGF receptors and also indirectly by inducing IL-6 (215). EGF induces cell motility and a mesenchymal morphology in OC cell lines and this effect of EGF is associated with the upregulation of N-cadherin and Vimentin (216). EGF also activates JAK2 and STAT3 signaling and changes both the abundance and localization of alpha6beta1 integrin in a manner that drives EMT induction and cancer cell migration (217). The EGF receptor ERBB3 has also been shown to regulate both Vimentin and E-cadherin *via* PI3K (218).

Hepatocyte growth factor can also induce EMT in OC cells (219). HGF, secreted by OC cells, has been shown to promote peritoneal implantation and HGF levels are found to be high in OC ascites as compared to benign fluids (219, 220). In addition to promoting OC migration, HGF also induces the migration of peritoneal MC by activating its receptor Met, leading to downstream Akt and ERK1/2 pathway activation.

Endothelin-1 has been shown to promote both EMT and chemoresistance in OC (221, 222). In resistant OC cells, ET-1 and Endothelin A receptor (ET(A)R) are upregulated and are also accompanied by enhanced MAPK and Akt phosphorylation and cell proliferation. When OC cells are treated with ET-1, expression of E-cadherin transcriptional repressors, including Snail, Slug, and Twist, as well as mesenchymal markers, such as Vimentin and N-cadherin, are all upregulated. Finally, analysis of tumor tissues derived from OC patients found that ET(A)R was overexpressed in resistant tumors and associated with an EMT phenotype (223). Hence, a number of cytokines and growth factors promote EMT in OC. These factors likely work together within the tumor microenvironment to promote and sustain dissemination and even resistance to therapy.

Most aggressive forms of EC have tumor cells that have migrated to nearby lymph nodes and have invaded through the myometrium of the uterus. Gene-expression microarrays followed by bioinformatic analysis revealed a potentially prominent role for the cytokine, TGF-β in promoting invasion through the induction of EMT (224). Other directors of the embryo implantation process such as FOS, MMP-9, MapK1, and RHOA were also associated with aggressive EC cases, which hints at a possible parallel between the molecular events associated with controlled trophoblast implantation and uncontrolled endometrial tumor invasion (225). In HEC1A and RL95-2 EC cell lines, EMT can be induced by TGF-β as tested at the morphological and molecular levels (224, 226). TGF-β can also act as a chemo attractant for these *in vitro* cell lines, increasing their invasive capacity (224). Co-treatment of Ishikawa cells with the cytokine, IL-6 and TGF-β resulted in messenchymal-like morphological changes that coincided with increased expression levels of the genes, Snail, N-cadherin, and Twist (227). Thus, TGF-β has been hypothesized to play a critical role in early invasion of EC through initiating the process associated with EMT. ERM/ETV5 (Ets family of transcription factors), is also upregulated in association with myometrial invasion (228). Overexpression of this particular transcription factor promotes cell migration and invasion and induces EMT by upregulating ZEB1 expression (229). In HEC1A EC cells, gene-expression microarray assays revealed Nidogen 1 (NID1) and Nuclear Protein 1(NUPR1) to be direct targets of the ETV5 transcription factor when it was stably expressed *in vitro* (230). At the invasive front, both NUPR1 and NID1 had similar expression levels to ETV5 (230). Knocking down NID1, a glycoprotein secreted by mesenchymal cells, in cells overexpressing ETV5 led to a significant decrease in cell invasion (230). Inhibiting NID1 in orthotopic EC models results in smaller tumors, an effect that is probably further enhanced by the microenvironment of the tumor (230). Additionally, inhibition of both NID1 and NUPR1 decreased the number of metastases (229). In HEC1A cells, ETV5 was shown to directly influence EMT by performing its function as a transcription factor and activating ZEB1. LPP, a protein implicated in cell-cell adhesion and cell motility is a transcriptional coactivator for other members of the transcription factor family of ETV5 (231). EMT induced by ETV5, led to localization of LPP from cell-cell contacts to focal adhesions (229). This accumulation of LPP at focal adhesions could lead to an amplification of extracellular signals and in turn its translocation to the nucleus, where in it could further activate ETV5, propagating its transcriptional activity and promoting persistent EC invasion through further EMT events.

Receptor activator of nuclear factor-κB (RANK) and its associated ligand RANKL, have been implicated in numerous physiological processes such as immune responses but also have been shown to be critical for the formation of lymph nodes (232). In EC tissue, RANK/RANKL expression is significantly higher and overexpression of RANK in EC cell lines, results in induction of EMT (233). CCL20 was found at increased levels in RANKL-treated RANK overexpressing cells. Furthermore, a neutralizing antibody targeting CCL20 could suppress EMT (233).

Autocrine motility factor (AMF) has also been implicated in EMT and, therefore, in promoting invasiveness and metastasis of endometrial carcinoma. Immunohistochemical analysis revealed high levels of AMF in EC tissue compared to normal endometrial tissue that showed a positive correlation with EMT markers (234). Silencing of AMF followed by gene-expression profiling showed altered expression of EMT mediators such as Snail (234). Additionally, treatment of EC cell lines with MAPK specific inhibitors downregulated EMT marker expression, suggesting that AMF promotes EMT in EC through the MAPK signaling pathway (234).

While some growth factors and cytokines such as TGF-β have well understood roles in both OC and EC progression through the promotion of EMT, more detailed study of EGF and HGF in EC are required before conclusions are drawn as to the strength of the role of those factors in the EMT process in the endometrium.

### Hypoxia and Oxidative Stress As Drivers of EMT in Ovarian and ECs

A hypoxic microenvironment is a common phenomenon existing in the central region of solid tumors due to insufficient penetration and diffusion of oxygen and nutrition (235). Under hypoxic conditions, the two subunits comprising hypoxia-inducible factor-1 (HIF-1) can form a functional complex, which in turn can activate the transcription of several genes whose expression correlates with cellular functions that promote aggressive tumor phenotypes (236).

Considerable evidence suggests that tumor hypoxia, followed by activation and stabilization of HIF-1 and its transcriptional targets, induces EMT, stem cell-like properties, neovascularization, altered energy metabolism, invasiveness, tumor cell spreading and intrinsic resistance to radiation and chemotherapy (237). Moreover, a meta-analysis from 25 studies of pathological and prognostic significance demonstrated that overexpression of HIF-1α is closely associated with high histological grade, advanced FIGO stage, lymph node metastasis and poor 5-year survival rate in patients with OC (238).

Hypoxic conditions can trigger an EMT program in OC cells through a series of mechanisms. For example, Notch signaling activated by hypoxic stimulus induces EMT, increased motility, and invasiveness through two various mechanisms that synergistically act to modulate Snail expression (239). First, it has been demonstrated that Notch directly upregulates Snail expression. Second, Notch potentiates HIF-1α recruitment to the *LOX* promoter which results in hypoxia-induced upregulation of LOX expression following by stabilization of Snail protein (239). Additionally, HIF-1α and LOX are highly expressed in OC tissues and significantly correlate with tumor grade and lymph node metastasis. The LOX and HIF-1α protein expression are markedly increased under hypoxic conditions and decreased after reoxygenation (240). Another study has linked HIF-1α with decreased E-cadherin in OC (241). Specifically, the hypoxic upregulation of HIF-1 and subsequent transcriptional induction of LOX and LOXL2 leads to the repression of E-cadherin and the subsequent induction of EMT. A recent study demonstrated that miR-210 is a master hypoxia sensor in OC (242). In response to hypoxia, miR-210 is upregulated in OC tissue as well as OC cell lines, and miR-210 mediates hypoxia-induced EMT by promoting Snail expression leading to inhibition of E-cadherin transcription. Additionally, miR-210 was found to increase the transcriptional activity of HIF-1, suggesting that a positive feedback loop may exist between miR-210 and HIF-1 that reciprocally modulates miR-210 release and thus sustains the function of miR-210 under hypoxia (243).

As has been described, chemokines and chemokine receptors mediate OC cell motility, invasion and metastasis (194). Chemokine receptor CCR7 expression is induced rapidly in OC cells in response to hypoxia, and participates in EMT induction, cell migration and invasion. Hypoxia synergizes with CCL21, the CCR7 ligand, to induce a strong upregulation of N-cadherin, Snail, and MMP-9 proteins (244).

Compelling experimental and clinical evidence indicates that ROS can also drive the dedifferentiation of cancer cells leading to EMT, invasion and metastasis (121). High levels of ROS in cancer cells are accumulated as a result of increased metabolic activity and mitochondrial dysfunction due to hypoxia and peroxisome activity (121). Levels of ROS can also be elevated by known ROS sources such as NADPH oxidases, cyclooxygenases, or lipoxygenases (121). Wang et al. have reported that ROS accumulation in OC cells leads to the HIF-1α-dependent induction of LOX which then represses E-cadherin expression (245).

A hypoxic microenvironment is also a substantial inducer of EMT in EC, as it tends to accompany rapidly growing solid malignancies and results from poor blood supply to surrounding healthy tissue. Directly or indirectly, HIF-1 has been shown to control Snail, ZEB and other regulators of EMT (246). In primary EC samples, HIF-1 was overexpressed in over 65% of cases (246). This elevated expression of HIF-1 coincided with increased expression of Twist and decreased levels of E-cadherin (246). It can be speculated that under low oxygen conditions HIF-1 regulates Twist expression by direct binding to its promoter and, therefore, promotes EMT and aggressiveness of EC.

Cancer cells, through activation of proteasome pathways, are capable of tolerating oxidative stress. REGϒ-associated proteasomes can degrade specific proteins like cell-cycle inhibitors in an ubiquitin and ATP-independent manner. In EC, mutant p53-R248Q can bind to the promoter of and upregulate the expression of REGϒ (247). Depletion of REGϒ in EC lines reduces cell proliferation, migration, and invasion where as expression of mutant p53-R248Q promotes EMT (247). Overexpression of p53-R248Q cannot restore REGϒ protein levels in REGϒ-depleted EC cells, hinting at an alternative mode in which the restoration of these cells’ malignant properties occurs (247). Insight from EC cells that are resistant to inhibitors of proteasomes, has led to the hypothesis that EMT in this environmental context is brought on by miR-200-ZEB1/ZEB2 protein regulation (248). p53-R248Q can also bind to the promoter of miR-130b, inhibiting its transcription and subsequently allowing ZEB1 to bring about the EMT phenotype (249). Interestingly, p53-R248Q has also been found to promote EMT in EC through the disruption of the p68-Drosha complex, which is responsible for the processing of miR-26a (250). Reduced miR-26a levels leads to overexpression of its downstream target EZH2, a promoter of EC tumor progression through EMT (250).

Although further research is required to fully understand the extent to which hypoxia influences the onset of EMT in the context of the endometrium, HIF-1 has been shown to significantly induce EMT in both EC and OC. Whether oxidative stress can also upregulate HIF-1 and bring about EMT in the endometrium also remains to be fully explored.

### Chemotherapy As a Driver of EMT in Ovarian Cancer

Despite initially successful multimodal therapy including tumor resection and platinum-based chemotherapy, tumor recurrence remains a major cause of mortality in OC patients (251). A growing body of evidence suggests that EMT and CSCs play important roles in the acquisition of chemoresistance in OC cells. Exposure to chemotherapeutics increases the percentage of CSCs within a tumor, the presence of which is correlated with metastatic progression, resistance to therapy and a poor clinical prognosis (30). Significantly, a paradigm that emerges from many recent studies is that residual cancer cells that become resistant to chemotherapy often undergo complete or partial EMT (252, 253). For example, in a recent study, primary OC cells treated with cisplatin experienced a loss of cell polarity, as well as an enrichment in cells expressing CSC markers at the protein and mRNA level (31). The expression of Snail, Slug, Twist, and MMP-2 was significantly enhanced in response to cisplatin and correlated with increased migration. In parallel, cell surface expression of CSC-like markers (CD44, α2 integrin subunit, CD117, CD133, EpCAM) and the expression of stem cell markers Nanog and Oct-4 were also significantly increased in response to cisplatin. These phenotypes were likely mediated by the ERK1/2 signaling pathway (252). Further, a pathway analysis associated with platinum resistance in OC demonstrated a strong association between EMT, stemness and resistance to platinum (254). Therefore, molecular link between platinum resistance, EMT and CSCs in OC has been supported by these data and it has been suggested that EMT may confer a selective survival advantage (254, 255).

## EMT and CTCs in Ovarian and ECs

In the context of tumor metastasis, recent studies have linked EMT to the onset of CTCs in OC and EC. Increasing evidence suggests that the presence of CTCs in bone marrow and in the peripheral blood of primary OC patients correlates with the presence of ascites and elevated CA-125 (256). Moreover, the detection of CTCs during follow-up occurs more often in older patients with platinum resistance and associates with impaired clinical outcome (256). Molecular profiling of CTCs from the same OC patients has shown that they express both stem cell (CD44, ALDH1A1, Nanog, Oct-4) and EMT (N-cadherin, Vimentin, Snail2, CD117, CD146) markers (257) suggesting that EMT stem or epithelial associated CTC traits are highly plastic.

The idea of tumor metastasis manifesting itself due to the dissemination of CTCs is a relatively new concept in the study of EC as previously the extent of myometrial infiltration and involvement of lymph nodes were used to define the likelihood of recurrent disease (258). As tumor cells can disseminate during early stages of tumor development, it is critical to utilize the most advanced technologies to detect CTCs in cancer patients to understand the exact mechanisms by which CTCs can contribute to metastatic disease. CTCs from EC patients have been immune-isolated using EpCAM and genes related to a number of key metastatic events (258). The EMT phenotype stood out as one of the main features when CTCs from EC were molecularly profiled (259). ETV5, Notch1, Snail, TGF-β1, ZEB1 and ZEB2, which are all genes associated with cellular plasticity, were significantly expressed in EC CTCs (258). *In vitro*, upregulation of ETV5 in the EC cell line HEC1A recapitulated the plasticity phenotype observed in the high-risk EC cases from which the CTC were derived (258). Microarray expression analysis of CTCs has also detected the presence of a protein component of the extracellular matrix that plays a role in tissue development, remodeling and repair, SPARC (259). CTCs derived from the cell line HEC1A contained enhanced levels of SPARC expression along with Fibronectin, a strong indicator of EMT (259). Overexpression of SPARC, stimulates the migratory activity of EC cells, a phenotype that is associated with CTCs (259). Thus, therapies targeting CTCs may be successful in halting the spread of metastasis from the endometrium.

It is apparent that CTCs possess stem cell-like traits in OC and EC most likely originating from EMT events in the presence or absence of therapeutics. In order to combat OC and EC metastasis directly, future work will need to address how the EMT process can be targeted to reduce the occurrence of proliferative and invasive forms of these carcinomas.

## Conclusion and Future Directions

In the last decade, EMT has emerged as a major driver of cancer progression in epithelial cancers. It allows cancer cells to detach and migrate, whilst also enabling the acquisition of CSC phenotypes. Cancers of the female reproductive tract, particularly OC and EC, also experience EMT. In OC this process is associated with disease progression, chemoresistance and the acquisition of CSC properties. Less is known about EMT in EC; however, it likely plays similar pro-tumorigenic roles in this disease. In order to combat EMT in cancer, one must fully understand how it is regulated. Several proteins, most notably members of the TGF-β superfamily, have been shown to drive EMT in cancer, by inducing epigenetic changes leading to transcriptional alterations. It is, however, very likely that other factors play an important role in the regulation of EMT and that cancer cells lack control mechanisms designed to counteract the EMT program. In contrast, the EMT events that occur during the development of the reproductive tract as well as the normal physiological functioning of adult organisms are highly regulated. By understanding the mechanisms by which EMT is regulated, and even reversed during normal development, we may reveal new targets for the treatment of OC and EC.

## Author Contributions

OB and MC conducted literature searches and wrote the review article. MJ designed and drew the diagram regarding EMT in OC and EC. L-MP reviewed and edited the manuscript.

## Conflict of Interest Statement

The authors declare that the research was conducted in the absence of any commercial or financial relationship that could be construed as a potential conflict of interest.

## References

[1] Evans T Sany O Pearmain P Ganesan R Blann A Sundar S. Differential trends in the rising incidence of endometrial cancer by type: data from a UK population-based registry from 1994 to 2006. Br J Cancer (2011) 104(9):1505–10. 10.1038/bjc.2011.68 21522151 PMC3101940

[2] Jemal A Bray F Center MM Ferlay J Ward E Forman D. Global cancer statistics. CA Cancer J Clin (2011) 61(2):69–90. 10.3322/caac.20107 21296855

[3] Elfayomy AK El Tarhouny SA. Ovarian volume assessment in relation to histologic findings and sex hormone levels in women with postmenopausal bleeding and thickened endometrium. Ann Saudi Med (2012) 32(6):588–92. 10.5144/0256-4947.2012.588 23396021 PMC6081122

[4] Jongen VH Sluijmer AV Heineman MJ. The postmenopausal ovary as an androgen-producing gland; hypothesis on the etiology of endometrial cancer. Maturitas (2002) 43(2):77–85. 10.1016/S0378-5122(02)00140-8 12385855

[5] Sozen H Vatansever D Iyibozkurt AC Topuz S Ozsurmeli M Salihoglu Y Clinicopathologic and survival analyses of synchronous primary endometrial and epithelial ovarian cancers. J Obstet Gynaecol Res (2015) 41(11):1813–9. 10.1111/jog.12826 26369625

[6] Merritt MA Cramer DW Molecular pathogenesis of endometrial and ovarian cancer. Cancer Biomark (2010) 9(1–6):287–305. 10.3233/CBM-2011-0167 22112481 PMC3822435

[7] Canadian Cancer Society’s Advisory Committee on Cancer Statistics. Canadian Cancer Statistics 2017. Toronto, ON: Canadian Cancer Society (2017).

[8] Prat J FIGO Committee on Gynecologic Oncology FIGO’s staging classification for cancer of the ovary, fallopian tube, and peritoneum: abridged republication. J Gynecol Oncol (2015) 26(2):87–9. 10.3802/jgo.2015.26.2.87 25872889 PMC4397237

[9] Edge SB Byrd DR Compton CC Fritz AG Greene FL Trotti A, editors. AJCC Cancer Staging Manual. 7th ed New York, NY: Springer (2010).

[10] Prat J FIGO Committee on Gynecologic Oncology Staging classification for cancer of the ovary, fallopian tube, and peritoneum: abridged republication of guidelines from the international federation of gynecology and obstetrics (FIGO). Obstet Gynecol (2015) 126(1):171–4. 10.1097/AOG.0000000000000917 26241270

[11] Ahmed N Stenvers KL. Getting to know ovarian cancer ascites: opportunities for targeted therapy-based translational research. Front Oncol (2013) 3:256. 10.3389/fonc.2013.00256 24093089 PMC3782691

[12] Smolle E Taucher V Haybaeck J. Malignant ascites in ovarian cancer and the role of targeted therapeutics. Anticancer Res (2014) 34(4):1553–61. 24692682

[13] Ozols RF. Challenges for chemotherapy in ovarian cancer. Ann Oncol (2006) 17(Suppl 5):v181–7. 10.1093/annonc/mdj978 16807453

[14] Shih Ie M Kurman RJ. Ovarian tumorigenesis: a proposed model based on morphological and molecular genetic analysis. Am J Pathol (2004) 164(5):1511–8. 10.1016/S0002-9440(10)63708-X 15111296 PMC1615664

[15] Meinhold-Heerlein I Fotopoulou C Harter P Kurzeder C Mustea A Wimberger P The new WHO classification of ovarian, fallopian tube, and primary peritoneal cancer and its clinical implications. Arch Gynecol Obstet (2016) 293(4):695–700. 10.1007/s00404-016-4035-8 26894303

[16] Kuo KT Mao TL Jones S Veras E Ayhan A Wang TL Frequent activating mutations of PIK3CA in ovarian clear cell carcinoma. Am J Pathol (2009) 174(5):1597–601. 10.2353/ajpath.2009.081000 19349352 PMC2671248

[17] Vereczkey I Serester O Dobos J Gallai M Szakacs O Szentirmay Z Molecular characterization of 103 ovarian serous and mucinous tumors. Pathol Oncol Res (2011) 17(3):551–9. 10.1007/s12253-010-9345-8 21136228

[18] Shin I Endometriosis-related ovarian cancer. An AACR special conference on Advances in Ovarian Cancer Research: Exploiting Vulnerabilities USA (2015):IA16.

[19] Nafisa W Pathology of the Ovary, Fallopian Tube and Peritoneum. London: Springer (2014). 520 p.

[20] Schwarz RF Ng CK Cooke SL Newman S Temple J Piskorz AM Spatial and temporal heterogeneity in high-grade serous ovarian cancer: a phylogenetic analysis. PLoS Med (2015) 12(2):e1001789. 10.1371/journal.pmed.1001789 25710373 PMC4339382

[21] Verhaak RG Tamayo P Yang JY Hubbard D Zhang H Creighton CJ Prognostically relevant gene signatures of high-grade serous ovarian carcinoma. J Clin Invest (2013) 123(1):517–25. 10.1172/JCI65833 23257362 PMC3533304

[22] Chen M Jin Y Bi Y Yin J Wang Y Pan L. A survival analysis comparing women with ovarian low-grade serous carcinoma to those with high-grade histology. Onco Targets Ther (2014) 7:1891–9. 10.2147/OTT.S67812 25342912 PMC4206388

[23] Reade CJ Mcvey RM Tone AA Finlayson SJ Mcalpine JN Fung-Kee-Fung M The fallopian tube as the origin of high grade serous ovarian cancer: review of a paradigm shift. J Obstet Gynaecol Can (2014) 36(2):133–40. 10.1016/S1701-2163(15)30659-9 24518912

[24] Wang Y Mang M Wang Y Wang L Klein R Kong B Tubal origin of ovarian endometriosis and clear cell and endometrioid carcinoma. Am J Cancer Res (2015) 5(3):869–79. 26045974 PMC4449423

[25] Moss EL Evans T Pearmain P Askew S Singh K Chan KK Should all cases of high-grade serous ovarian, tubal, and primary peritoneal carcinomas be reclassified as tubo-ovarian serous carcinoma? Int J Gynecol Cancer (2015) 25(7):1201–7. 10.1097/Igc.0000000000000477 26035124

[26] Pereira A Mendizabal E De Leon J Perez-Medina T Magrina JF Magtibay PM Peritoneal carcinomatosis: a malignant disease with an embryological origin? Surg Oncol (2015) 24(3):305–11. 10.1016/j.suronc.2015.06.002 26141556

[27] Gurung A Hung T Morin J Gilks CB. Molecular abnormalities in ovarian carcinoma: clinical, morphological and therapeutic correlates. Histopathology (2013) 62(1):59–70. 10.1111/his.12033 23240670

[28] Wang V Li C Lin M Welch W Bell D Wong YF Ovarian cancer is a heterogeneous disease. Cancer Genet Cytogen (2005) 161(2):170–3. 10.1016/j.cancergencyto.2004.12.014 16102589

[29] Meacham CE Morrison SJ. Tumour heterogeneity and cancer cell plasticity. Nature (2013) 501(7467):328–37. 10.1038/nature12624 24048065 PMC4521623

[30] Tomao F Papa A Strudel M Rossi L Lo Russo G Benedetti Panici P Investigating molecular profiles of ovarian cancer: an update on cancer stem cells. J Cancer (2014) 5(5):301–10. 10.7150/jca.8610 24723972 PMC3982176

[31] Abubaker K Latifi A Luwor R Nazaretian S Zhu H Quinn MA Short-term single treatment of chemotherapy results in the enrichment of ovarian cancer stem cell-like cells leading to an increased tumor burden. Mol Cancer (2013) 12:24. 10.1186/1476-4598-12-24 23537295 PMC3668985

[32] Creasman WT Odicino F Maisonneuve P Beller U Benedet JL Heintz AP Carcinoma of the corpus uteri. J Epidemiol Biostat (2001) 6(1):47–86. 11385776

[33] Obel JC Friberg G Fleming GF. Chemotherapy in endometrial cancer. Clin Adv Hematol Oncol (2006) 4(6):459–68. 16981669

[34] Lax SF Kurman RJ A dualistic model for endometrial carcinogenesis based on immunohistochemical and molecular genetic analyses. Verh Dtsch Ges Pathol (1997) 81:228–32. 9474874

[35] Cancer Genome Atlas Research Network Kandoth C Schultz N Cherniack AD Akbani R Liu Y Integrated genomic characterization of endometrial carcinoma. Nature (2013) 497(7447):67–73. 10.1038/nature12113 23636398 PMC3704730

[36] Cheung LW Hennessy BT Li J Yu S Myers AP Djordjevic B High frequency of PIK3R1 and PIK3R2 mutations in endometrial cancer elucidates a novel mechanism for regulation of PTEN protein stability. Cancer Discov (2011) 1(2):170–85. 10.1158/2159-8290.CD-11-0039 21984976 PMC3187555

[37] Levine RL Cargile CB Blazes MS Van Rees B Kurman RJ Ellenson LH. PTEN mutations and microsatellite instability in complex atypical hyperplasia, a precursor lesion to uterine endometrioid carcinoma. Cancer Res (1998) 58(15):3254–8. 9699651

[38] Mcconechy MK Ding JR Cheang MCU Wiegand KC Senz J Tone AA Use of mutation profiles to refine the classification of endometrial carcinomas. J Pathol (2012) 228(1):20–30. 10.1002/path.4056 22653804 PMC3939694

[39] Byron SA Gartside M Powell MA Wellens CL Gao F Mutch DG FGFR2 point mutations in 466 endometrioid endometrial tumors: relationship with MSI, KRAS, PIK3CA, CTNNB1 mutations and clinicopathological features. PLoS One (2012) 7(2):e30801. 10.1371/journal.pone.0030801 22383975 PMC3285611

[40] Urick ME Rudd ML Godwin AK Soroi D Merino M Bell DW PIK3R1 (p85α) is somatically mutated at high frequency in primary endometrial cancer. Cancer Res (2011) 71:4061–7. 10.1158/0008-5472.CAN-11-0549 21478295 PMC3117071

[41] Kuhn E Wu RC Guan B Wu G Zhang J Wang Y Identification of molecular pathway aberrations in uterine serous carcinoma by genome-wide analyses. J Natl Cancer Inst (2012) 104(19):1503–13. 10.1093/jnci/djs345 22923510 PMC3692380

[42] Le Gallo M O’hara AJ Rudd ML Urick ME Hansen NF O’neil NJ Exome sequencing of serous endometrial tumors identifies recurrent somatic mutations in chromatin-remodeling and ubiquitin ligase complex genes. Nat Genet (2012) 44(12):1310–5. 10.1038/ng.2455 23104009 PMC3515204

[43] Kuhn E Ayhan A Bahadirli-Talbott A Zhao C Shih I-M Molecular characterization of undifferentiated carcinoma associated with endometriod carcinoma. Am J Surg Pathol (2014) 38:660–5. 10.1097/PAS.0000000000000166 24451280

[44] Kim YS Yi BR Kim NH Choi KC. Role of the epithelial-mesenchymal transition and its effects on embryonic stem cells. Exp Mol Med (2014) 46:e108. 10.1038/emm.2014.44 25081188 PMC4150931

[45] Lamouille S Xu J Derynck R Molecular mechanisms of epithelial-mesenchymal transition. Nat Rev Mol Cell Biol (2014) 15(3):178–96. 10.1038/nrm3758 24556840 PMC4240281

[46] Kudo-Saito C Shirako H Takeuchi T Kawakami Y Cancer metastasis is accelerated through immunossupression during Snail-induced EMT of cancer cells. Cancer Cell (2009) 15:195–206. 10.1016/j.ccr.2009.01.023 19249678

[47] Iwatsuki M Mimori K Yokobori T Ishi H Beppu T Nakamori S Epithelial-mesenchymal transition in cancer development and its clinical significance. Cancer Sci (2010) 101(2):293–9. 10.1111/j.1349-7006.2009.01419.x 19961486 PMC11159985

[48] Singh A Settleman J. EMT, cancer stem cells and drug resistance: an emerging axis of evil in the war on cancer. Oncogene (2010) 29(34):4741–51. 10.1038/onc.2010.215 20531305 PMC3176718

[49] Thiery JP Epithelial-mesenchymal transitions in tumor progression. Nat Rev Cancer (2002) 2:442–54. 10.1038/nrc822 12189386

[50] Mirantes C Espinosa I Ferrer I Dolcet X Prat J Matias-Guiu X. Epithelial-to-mesenchymal transition and stem cells in endometrial cancer. Hum Pathol (2013) 44(10):1973–81. 10.1016/j.humpath.2013.04.009 23845467

[51] Bhowmick NA Neilson EG Moses HL. Stromal fibroblasts in cancer initiation and progression. Nature (2004) 432(7015):332–7. 10.1038/nature03096 15549095 PMC3050735

[52] Auersperg N Wong AS Choi KC Kang SK Leung PC. Ovarian surface epithelium: biology, endocrinology, and pathology. Endocr Rev (2001) 22(2):255–88. 10.1210/edrv.22.2.0422 11294827

[53] Mora JM Fenwick MA Castle L Baithun M Ryder TA Mobberley M Characterization and significance of adhesion and junction-related proteins in mouse ovarian follicles. Biol Reprod (2012) 86(5):1–14. 10.1095/biolreprod.111.096156 22321830

[54] Sawyer HR Smith P Heath DA Juengel JL Wakefield SJ Mcnatty KP Formation of ovarian follicles during fetal development in sheep. Biol Reprod (2002) 66(4):1134–50. 10.1095/biolreprod66.4.1134 11906935

[55] Zheng W Zhang H Gorre N Risal S Shen Y Liu K. Two classes of ovarian primordial follicles exhibit distinct developmental dynamics and physiological functions. Hum Mol Genet (2014) 23(4):920–8. 10.1093/hmg/ddt486 24087793 PMC3900105

[56] Mork L Maatouk DM Mcmahon JA Guo JJ Zhang P Mcmahon AP Temporal differences in granulosa cell specification in the ovary reflect distinct follicle fates in mice. Biol Reprod (2012) 86(2):37. 10.1095/biolreprod.111.095208 21976597 PMC3290667

[57] Eppig JJ Handel MA Origin of granulosa cells clarified and complexified by waves. Biol Reprod (2012) 86:34 10.1095/biolreprod.111.096651 21976596

[58] Childs AJ Mcneilly AS Epithelial-to-mesenchymal transition in granulosa cells: a key to activation of follicle growth? Biol Reprod (2012) 86(5):152, 1–2. 10.1095/biolreprod.112.100156 22402964

[59] Kezele P Nilsson EE Skinner MK. Keratinocyte growth factor acts as a mesenchymal factor that promotes ovarian primordial to primary follicle transition. Biol Reprod (2005) 152:1–2. 10.1095/biolreprod.112.100156 16000551

[60] Wang ZP Mu XY Guo M Wang YJ Teng Z Mao GP Transforming growth factor-beta signaling participates in the maintenance of the primordial follicle pool in the mouse ovary. J Biol Chem (2014) 289(12):8299–311. 10.1074/jbc.M113.532952 24515103 PMC3961657

[61] Knight PG Glister C. TGF-beta superfamily members and ovarian follicle development. Reproduction (2006) 132(2):191–206. 10.1530/rep.1.01074 16885529

[62] Kristensen SG Andersen K Clement CA Franks S Hardy K Andersen CY. Expression of TGF-beta superfamily growth factors, their receptors, the associated SMADs and antagonists in five isolated size-matched populations of pre-antral follicles from normal human ovaries. Mol Hum Reprod (2014) 20:293–308. 10.1093/molehr/gat089 24270394

[63] Zhu Y Nilsson M Sundfeldt K Phenotypic plasticity of the ovarian surface epithelium: TGF-β1 induction of epithelial to mesenchymal transition (EMT) *in vitro*. Endocrinology (2010) 151:5497–505. 10.1210/en.2010-0486 20844000

[64] Miyazono K Transforming growth factor-beta signaling in epithelial-mesenchymal transition and progression of cancer. Proc Jpn Acad Ser B Phys Biol Sci (2009) 85(8):314–23. 10.2183/pjab.85.314 PMC3621568 19838011

[65] Garg M Epithelial-mesenchymal transition – activating transcription factors – multifunctional regulators in cancers. World J Stem Cells (2013) 5:188–95. 10.4252/wjsc.v5.i4.188 24179606 PMC3812522

[66] Zhang YE. Non-Smad pathways in TGF-beta signaling. Cell Res (2009) 19(1):128–39. 10.1038/cr.2008.328 19114990 PMC2635127

[67] Cheng JC Chang HM Fang L Sun YP Leung PC TGF-β1 up-regulates connective tissue growth factor expression in human granulosa cells through Smad and ERK1/2 signaling pathways. PLoS One (2015) 10:e0126532 10.1371/journal.pone.0126532 25955392 PMC4425519

[68] Patel IS Madan P Getsios S Bertrand MA Maccalman CD Cadherin switching in ovarian cancer progression. Int J Cancer (2003) 106(2):172–7. 10.1002/ijc.11086 12800191

[69] Czernobilsky B Moll R Levy R Franke WW. Co-expression of cytokeratin and vimentin filaments in mesothelial, granulosa and rete ovarii cells of the human ovary. Eur J Cell Biol (1985) 37:175–90. 3896804

[70] Mclennan CE Rydell AH Extent of endometrial shedding during normal menstruation. Obstet Gynecol (1965) 26(5):605–21. 5855347

[71] Jabbour HN Kelly RW Fraser HM Critchley HO. Endocrine regulation of menstruation. Endocr Rev (2006) 27(1):17–46. 10.1210/er.2004-0021 16160098

[72] Buckley CH Fox H The normal endometrium as seen in biopsy material. Biopsy Pathology of the Endometrium. London: Lippincott Williams & Wilkins (1989). p. 30–47.

[73] Yang J Weinberg RA. Epithelial-mesenchymal transition: at the crossroads of development and tumor metastasis. Dev Cell (2008) 14(6):818–29. 10.1016/j.devcel.2008.05.009 18539112

[74] Orvis GD Behringer RR Cellular mechanisms of Mullerian duct formation in the mouse. Dev Biol (2007) 306(2):493–504. 10.1016/j.ydbio.2007.03.027 17467685 PMC2730733

[75] Klattig J Englert C The Mullerian duct: recent insights into its development and regression. Sex Dev (2007) 1(5):271–8. 10.1159/000108929 18391537

[76] Mericskay M Kitajewski J Sassoon D. Wnt5a is required for proper epithelial-mesenchymal interactions in the uterus. Development (2004) 131(9):2061–72. 10.1242/dev.01090 15073149

[77] Miller C Sassoon DA. Wnt-7a maintains appropriate uterine patterning during the development of the mouse female reproductive tract. Development (1998) 125(16):3201–11. 9671592 10.1242/dev.125.16.3201

[78] Stewart CA Wang Y Bonilla-Claudio M Martin JF Gonzalez G Taketo MM CTNNB1 in mesenchyme regulates epithelial cell differentiation during Mullerian duct and postnatal uterine development. Mol Endocrinol (2013) 27(9):1442–54. 10.1210/me.2012-1126 23904126 PMC3753424

[79] Uchida H Maruyama T Nishikawa-Uchida S Oda H Miyazaki K Yamasaki A Studies using an in vitro model show evidence of involvement of epithelial-mesenchymal transition of human endometrial epithelial cells in human embryo implantation. J Biol Chem (2012) 287(7):4441–50. 10.1074/jbc.M111.286138 22174415 PMC3281640

[80] Lee KY Demayo FJ Animal models of implantation. Reproduction (2004) 128(6):679–95. 10.1530/rep.1.00340 15579585

[81] Paria BC Zhao X Das SK Dey SK Yoshinaga K. Zonula occludens-1 and E-cadherin are coordinately expressed in the mouse uterus with the initiation of implantation and decidualization. Dev Biol (1999) 208(2):488–501. 10.1006/dbio.1999.9206 10191061

[82] Zhang XH Liang X Liang XH Wang TS Qi QR Deng WB The mesenchymal-epithelial transition during in vitro decidualization. Reprod Sci (2013) 20(4):354–60. 10.1177/1933719112472738 23302397 PMC4077516

[83] Renjini AP Titus S Narayan P Murali M Jha RK Laloraya M. STAT3 and MCL-1 associate to cause a mesenchymal epithelial transition. J Cell Sci (2014) 127(Pt 8):1738–50. 10.1242/jcs.138214 24481815

[84] Murdoch WJ Ovulatory factor in ovarian carcinogenesis. Adv Exp Med Biol (2008) 622:119–28. 10.1007/978-0-387-68969-2_10 18546623

[85] Ahmed N Thompson EW Quinn MA. Epithelial-mesenchymal interconversions in normal ovarian surface epithelium and ovarian carcinomas: an exception to the norm. J Cell Physiol (2007) 213(3):581–8. 10.1002/jcp.21240 17708542

[86] Brown TJ Shathasivam P Maintaining mesenchymal properties of ovarian surface epithelial cells: a potential early protective role for TGF-beta in ovarian carcinogenesis. Endocrinology (2010) 151(11):5092–4. 10.1210/en.2010-0938 20962057

[87] Gamwell LF Collins O Vanderhyden BC The mouse ovarian surface epithelium contains a population of LY6A (SCA-1) expressing progenitor cells that are regulated by ovulation-associated factors. Biol Reprod (2012) 87:4 10.1095/biolreprod.112.100347 22914315

[88] Ahmed N Maines-Bandiera S Quinn MA Unger WG Dedhar S Auersperg N Molecular pathways regulating EGF-induced epithelia-mesenchymal transition in human ovarian surface epithelium. Am J Physiol Cell Physiol (2006) 290:1532–42. 10.1152/ajpcell.00478.2005 16394028

[89] Critchley HO Kelly RW Brenner RM Baird DT The endocrinology of menstruation – a role for the immune system. Clin Endocrinol (Oxf) (2001) 55(6):701–10. 10.1046/j.1365-2265.2001.01432.x 11895208

[90] Ferenczy A. Studies on the cytodynamics of human endometrial regeneration. II. Transmission electron microscopy and histochemistry. Am J Obstet Gynecol (1976) 124(6):582–95. 10.1016/0002-9378(76)90059-4 943943

[91] Gargett CE Chan RW Schwab KE. Hormone and growth factor signaling in endometrial renewal: role of stem/progenitor cells. Mol Cell Endocrinol (2008) 288(1–2):22–9. 10.1016/j.mce.2008.02.026 18403104

[92] Patterson A Zhang L Arango N Teixeira J Pru J Mesenchymal-to-epithelial transition contributes to endometrial regeneration following natural and articial decidualization. Stem Cells Dev (2013) 22(6):964–74. 10.1089/scd.2012.0435 23216285 PMC3585744

[93] Cousins FL Murray A Esnal A Gibson DA Critchley HOD Saunders PTK Evidence from a mouse model that epithelial cell migration and mesenchymal-epithelial transition contribute to rapid restoration of uterine tissue integrity during menstruation. PLoS One (2014) 9:1 10.1371/journal.pone.0086378 PMC3899239 24466063

[94] Tamai K Togashi K Ito T Morisawa N Fujiwara T Koyama T. MR imaging findings of adenomyosis: correlation with histopathologic features and diagnostic pitfalls. Radiographics (2005) 25(1):21–40. 10.1148/rg.251045060 15653584

[95] Ferenczy A. Pathophysiology of adenomyosis. Hum Reprod Update (1998) 4(4):312–22. 10.1093/humupd/4.4.312 9825847

[96] Wang PH Su WH Sheu BC Liu WM. Adenomyosis and its variance: adenomyoma and female fertility. Taiwan J Obstet Gynecol (2009) 48(3):232–8. 10.1016/S1028-4559(09)60295-3 19797011

[97] Schmalhofer O Brabletz S Brabletz T E-cadherin, beta-catenin, and ZEB1 in malignant progression of cancer. Cancer Metastasis Rev (2009) 28(1–2):151–66. 10.1007/s10555-008-9179-y 19153669

[98] Oh S Shin J Kim T Lee H Yoo J Ahn J β-catenin activation contributes to the pathogenesis of adenomyosis through epithelial-mesenchymal transition. J Pathol (2013) 231(2):210–22. 10.1002/path.4224 23784889 PMC4105844

[99] Chen YJ Li HY Huang CH Twu NF Yen MS Wang PH Oestrogen-induced epithelial-mesenchymal transition of endometrial epithelial cells contributes to the development of adenomyosis. J Pathol (2010) 222(3):261–70. 10.1002/path.2761 20814901

[100] Proestling K Birner P Gamperl S Nirtl N Marton E Yerlikaya G Enhanced epithelial to mesenchymal transition (EMT) and upregulated MYC in ectopic lesions contribute independently to endometriosis. Reprod Biol Endocrinol (2015) 13:75. 10.1186/s12958-015-0063-7 26198055 PMC4511248

[101] Vargha R Bender TO Riesenhuber A Endemann M Kratochwill K Aufricht C. Effects of epithelial-to-mesenchymal transition on acute stress response in human peritoneal mesothelial cells. Nephrol Dial Transplant (2008) 23(11):3494–500. 10.1093/ndt/gfn353 18577533

[102] Peinado H Del Carmen Iglesias-De La Cruz M Olmeda D Csiszar K Fong KS Vega S A molecular role for lysyl oxidase-like 2 enzyme in snail regulation and tumor progression. EMBO J (2005) 24(19):3446–58. 10.1038/sj.emboj.7600781 16096638 PMC1276164

[103] Moreno-Bueno G Salvador F Martin A Floristan A Cuevas EP Santos V Lysyl oxidase-like 2 (LOXL2), a new regulator of cell polarity required for metastatic dissemination of basal-like breast carcinomas. EMBO Mol Med (2011) 3(9):528–44. 10.1002/emmm.201100156 21732535 PMC3377095

[104] Talbi S Hamilton AE Vo KC Tulac S Overgaard MT Dosiou C Molecular phenotyping of human endometrium distinguishes mensrual cycle phases and underlying biological processes in normo-ovulatory women. Endocrinology (2006) 147:797–816. 10.1210/en.2005-1076 16306079

[105] Savaris RF Hamilton AE Lessey BA Giudice LC. Endometrial gene expression in early pregnancy: lessons from human ectopic pregnancy. Reprod Sci (2008) 15(8):797–816. 10.1177/1933719108317585 18591649 PMC2882188

[106] Ruiz LA Dutil J Ruiz A Fourquet J Abac S Laboy J Single-nucleotide polymorphisms in the lysyl oxidase-like protein 4 and complement component 3 genes are associated with increased risk for endometriosis and endometriosis-associated infertility. Fertil Steril (2011) 96(2):512–5. 10.1016/j.fertnstert.2011.06.001 21733505 PMC3143268

[107] Ruiz LA Baez-Vega PM Ruiz A Peterse DP Monteiro JB Bracero N Dysregulation of lysyl oxidase expression in lesions and endometrium of women with endometriosis. Reprod Sci (2015) 22(12):1496–508. 10.1177/1933719115585144 25963914 PMC5933196

[108] Konno R Fujiwara H Netsu S Odagiri K Shimane M Nomura H Gene expression profiling of the rat endometriosis model. Am J Reprod Immunol (2007) 58(4):330–43. 10.1111/j.1600-0897.2007.00507.x 17845203

[109] Lin HH Liao CJ Lee YC Hu KH Meng HW Chu ST Lipcalin-2-induced cytokine production enhances endometrial carcinoma cell survival and migration. Int J Biol Sci (2011) 7(1):74–86. 10.7150/ijbs.7.74 21278918 PMC3030144

[110] Liao CJ Li PT Lee YC Li SH Chu ST Lipcalin 2 induces the epithelial-mesenchymal transition in stressed endometrial epithelial cells: possible correlation with endometriosis development in a mouse model. Reproduction (2014) 147:2 10.1530/REP-13-0236 24194573

[111] Zheng QM Lu JJ Zhao J Wei X Wang L Liu PS. Periostin facilitates the epithelial-mesenchymal transition of endometrial epithelial cells through ILK-Akt signaling pathway. Biomed Res Int (2016) 2016:9842619. 10.1155/2016/9842619 27034956 PMC4808541

[112] Demir AY Groothuis PG Nap AW Punyadeera C De Goeij AF Evers JL Menstrual effluent induces epithelial-mesenchymal transitions in mesothelial cells. Hum Reprod (2004) 19(1):21–9. 10.1093/humrep/deh042 14688152

[113] Matsuzaki S Darcha C Epithelial to mesenchymal transition-like and mesenchymal to epithelial transition-like processes might be involved in the pathogenesis of pelvic endometriosis. Hum Reprod (2012) 27(3):712–21. 10.1093/humrep/der442 22215621

[114] Liu Q Zhang Y Mao H Chen W Luo N Zhou Q A crosstalk between the Smad and JNK signaling in the TGF-β induced epithelial-mesenchymal transition in rat peritoneal mesothelial cells. PLoS One (2012) 7:2 10.1371/journal.pone.0032009 PMC3288060 22384127

[115] Young VJ Brown JK Saunders PT Duncan WC Horne AW. The peritoneum is both a source and target of TGF-β in women with endometriosis. PLoS One (2014) 9:9. 10.1371/journal.pone.0106773 25207642 PMC4160207

[116] King SM Hilliard TS Wu LY Jaffe RC Fazleabas AT Burdette JE The impact of ovulation on fallopian tube epithelial cells: evaluation three hypotheses connecting ovulation and serous ovarian cancer. Endocr Relat Cancer (2011) 7(2):627–42. 10.1530/ERC-11-0107 PMC3638747 21813729

[117] Seidman JD The presence of mucosal iron in the fallopian tube supports the “incessant menstruation hypothesis” for ovarian carcinoma. Int J Gynecol Pathol (2013) 32(5):454–8. 10.1097/PGP.0b013e31826f5ce2 23896711

[118] Kuhn E Kurman RJ Vang R Sehdev AS Han G Soslow R TP53 mutations in serous tubal intraepithelial carcinoma and concurrent pelvic high-grade serous carcinoma – evidence supporting the clonal relationship of the two lesions. J Pathol (2012) 226(3):421–6. 10.1002/path.3023 21990067 PMC4782784

[119] Iwanicki MP Chen HY Iavarone C Zervantonakis IK Muranen T Novak M Mutant p53 regulates ovarian cancer transformed phenotypes through autocrine matrix deposition. JCI Insight (2016) 1:10. 10.1172/jci.insight.86829 27482544 PMC4963159

[120] Hanahan D Weinberg RA Hallmarks of cancer: the next generation. Cell (2011) 144(5):646–74. 10.1016/j.cell.2011.02.013 21376230

[121] Fiaschi T Chiarugi P. Oxidative stress, tumor microenvironment, and metabolic reprogramming: a diabolic liaison. Int J Cell Biol (2012) 2012:762825. 10.1155/2012/762825 22666258 PMC3361160

[122] Mahalingaiah PK Ponnusamy L Singh KP. Chronic oxidative stress leads to malignant transformation along with acquisition of stem cell characteristics, and epithelial to mesenchymal transition in human renal epithelial cells. J Cell Physiol (2015) 230(8):1916–28. 10.1002/jcp.24922 25546616

[123] Acs G Serous and mucinous borderline (low malignant potential) tumors of the ovary. Am J Clin Pathol (2005) 123(Suppl):S13–57. 16100867 10.1309/J6PXXK1HQJAEBVPM

[124] Morice P Uzan C Fauvet R Gouy S Duvillard P Darai E Borderline ovarian tumour: pathological diagnostic dilemma and risk factors for invasive or lethal recurrence. Lancet Oncol (2012) 13(3):E103–15. 10.1016/S1470-2045(11)70288-1 22381933

[125] Cheng JC Auersperg N Leung PC. EGF-induced EMT and invasiveness in serous borderline ovarian tumor cells: a possible step in the transition to low-grade serous carcinoma cells? PLoS One (2012) 7(3):e34071. 10.1371/journal.pone.0034071 22479527 PMC3316602

[126] Cheng JC Auersperg N Leung PC. TGF-beta induces serous borderline ovarian tumor cell invasion by activating EMT but triggers apoptosis in low-grade serous ovarian carcinoma cells. PLoS One (2012) 7(8):e42436. 10.1371/journal.pone.0042436 22905131 PMC3419689

[127] Lengyel E Ovarian cancer development and metastasis. Am J Pathol (2010) 177(3):1053–64. 10.2353/ajpath.2010.100105 20651229 PMC2928939

[128] Chien J Shridhar V Mariani A Evidence for extra-origin and peritoneal metastases that precede ovarian carcinomas in high-grade serous ovarian cancer. An AACR Special Conference on Advances in Ovarian Cancer Research: Exploiting Vulnerability USA (2015):PR08.

[129] Yeung TL Leung CS Yip KP Au Yeung CL Wong ST Mok SC. Cellular and molecular processes in ovarian cancer metastasis. A review in the theme: cell and molecular processes in cancer metastasis. Am J Physiol Cell Physiol (2015) 309(7):C444–56. 10.1152/ajpcell.00188.2015 26224579 PMC4593771

[130] Pradeep S Kim SW Wu SY Nishimura M Chaluvally-Raghavan P Miyake T Hematogenous metastasis of ovarian cancer: rethinking mode of spread. Cancer Cell (2014) 26(1):77–91. 10.1016/j.ccr.2014.05.002 25026212 PMC4100212

[131] Moreno-Bueno G Portillo F Cano A Transcriptional regulation of cell polarity in EMT and cancer. Oncogene (2008) 27(55):6958–69. 10.1038/onc.2008.346 19029937

[132] Yoshida J Horiuchi A Kikuchi N Hayashi A Osada R Ohira S Changes in the expression of E-cadherin repressors, Snail, Slug, SIP1, and Twist, in the development and progression of ovarian carcinoma: the important role of Snail in ovarian tumorigenesis and progression. Med Mol Morphol (2009) 42(2):82–91. 10.1007/s00795-008-0436-5 19536615

[133] Montserrat N Mozos A Llobet D Dolcet X Pons C De Herreros AG Epithelial to mesenchymal transition in early stage endometrioid endometrial carcinoma. Hum Pathol (2012) 43(5):632–43. 10.1016/j.humpath.2011.06.021 21940036

[134] Saegusa M Hasimura M Kuwata T Okayasu I Requirement of the Akt/β-catenin pathway for uterine carcinoscarcoma genesis modulating E-cadherin expression through the transactivation of Slug. Am J Pathol (2009) 174:2107–15. 10.2353/ajpath.2009.081018 19389926 PMC2684176

[135] Lau MT Klausen C Leung PC E-cadherin inhibits tumor cell growth by suppressing PI3K/Akt signaling via beta-catenin-Egr1-mediated PTEN expression. Oncogene (2011) 30(24):2753–66. 10.1038/onc.2011.6 21297666

[136] Inoue H Takahashi H Hashimura M Eshima K Akiya M Matsumoto T Cooperation of Sox4 with beta-catenin/p300 complex in transcriptional regulation of the Slug gene during divergent sarcomatous differentiation in uterine carcinosarcoma. BMC Cancer (2016) 16:53 10.1186/s12885-016-2090-y 26841870 PMC4739330

[137] Bian YD Chang XW Liao Y Wang JY Li YR Wang K Promotion of epithelial-mesenchymal transition by Frizzled2 is involved in the metastasis of endometrial cancer. Oncol Rep (2016) 36(2):803–10. 10.3892/or.2016.4885 27373314

[138] Liu L Zhang J Yang XM Fang C Xu HL Xi XW. SALL4 as an epithelial-mesenchymal transition and drug resistance inducer through the regulation of c-Myc in endometrial cancer. PLoS One (2015) 10(9):e0138515. 10.1371/journal.pone.0138515 26407074 PMC4583418

[139] Gumireddy K Li AP Gimotty PA Klein-Szanto AJ Showe LC Katsaros D KLF17 is a negative regulator of epithelial-mesenchymal transition and metastasis in breast cancer. Nat Cell Biol (2009) 11(11):1297–304. 10.1038/ncb1974 19801974 PMC2784164

[140] Cai XD Zhou YB Huang LX Zeng QL Zhang LJ Wang QQ Reduced expression of Kruppel-like factor 17 is related to tumor growth and poor prognosis in lung adenocarcinoma. Biochem Biophys Res Commun (2012) 418(1):67–73. 10.1016/j.bbrc.2011.12.129 22240024

[141] Liu FY Deng YL Li Y Zeng D Zhou ZZ Tian DA Down-regulated KLF17 expression is associated with tumor invasion and poor prognosis in hepatocellular carcinoma. Med Oncol (2013) 30:1. 10.1007/s12032-012-0425-3 23325444

[142] Dong PX Kaneuchi M Xiong Y Cao LP Cai MY Liu XS Identification of KLF17 as a novel epithelial to mesenchymal transition inducer via direct activation of TWIST1 in endometrioid endometrial cancer. Carcinogenesis (2014) 35(4):760–8. 10.1093/carcin/bgt369 24220291

[143] Chen Z Wang Y Liu W Zhao G Lee S Balogh A Doxocycline inducible Krüppel-like factor 4 lentiviral vector mediates mesenchymal to epithelial transition in ovarian cancer cells. PLoS One (2014) 9:e105331 10.1371/journal.pone.0105331 25137052 PMC4138168

[144] Bao W Qiu H Yang T Luo X Zhang H Wan X. Upregulation of TrkB promotes epithelial-mesenchymal transition and anoikis resistance in endometrial carcinoma. PLoS One (2013) 8(7):e70616. 10.1371/journal.pone.0070616 23936232 PMC3728299

[145] Douma S Van Laar T Zevenhoven J Meuwissen R Van Garderen E Peeper DS. Suppression of anoikis and induction of metastasis by the neurotrophic receptor TrkB. Nature (2004) 430(7003):1034–9. 10.1038/nature02765 15329723

[146] Zheng W Dai Q Tao P Sun A Wang Y Bao L Overexpression of tyrosine kinase receptor B promotes metastasis of ovarian serous adenocarcinoma by lymphangiogenesis. Tumori (2011) 97(6):756–61. 10.1700/1018.11093 22322843

[147] Altenberg B Greulich KO. Genes of glycolysis are ubiquitously overexpressed in 24 cancer classes. Genomics (2004) 84(6):1014–20. 10.1016/j.ygeno.2004.08.010 15533718

[148] Zhao MY Fang WY Wang Y Guo SQ Shu LY Wang LJ Enolase-1 is a therapeutic target in endometrial carcinoma. Oncotarget (2015) 6(17):15610–27. 10.18632/oncotarget.3639 25951350 PMC4558174

[149] Tsai HC Baylin SB. Cancer epigenetics: linking basic biology to clinical medicine. Cell Res (2011) 21(3):502–17. 10.1038/cr.2011.24 21321605 PMC3193419

[150] Li Y Fang Y Liu Y Yang X MicroRNAs in ovarian function and disorders. J Ovarian Res (2015) 8:51 10.1186/s13048-015-0162-2 26232057 PMC4522283

[151] Castilla MA Moreno-Bueno G Romero-Perez L Van De Vijver K Biscuola M Lopez-Garcia MA MicroRNA signature of the epithelial-mesenchymal transition in endometrial carcinosarcoma. J Pathol (2011) 223:72–80. 10.1002/path.2802 21125666

[152] Filipowicz W Bhattacharyya SN Sonenberg N. Mechanisms of post-transcriptional regulation by microRNAs: are the answers in sight? Nat Rev Genet (2008) 9(2):102–14. 10.1038/nrg2290 18197166

[153] Dong P Kaneuchi M Watari H Hamada J Sudo S Ju J MicroRNA-194 inhibits epithelial to mesenchymal transition of endometrial cancer cells by targeting oncogene BMI-1. Mol Cancer (2011) 10:99. 10.1186/1476-4598-10-99 21851624 PMC3173388

[154] Konno Y Dong P Xiong Y Suzuki F Lu J Cai M MicroRNA-101 targets EZH3, MCL-1 and FOS to suppress proliferation, invasion and stem cell-like phenotype of aggressive endometrial cancer cells. Oncotarget (2014) 5:6049–62. 10.18632/oncotarget.2157 25153722 PMC4171612

[155] Liu P Wang C Ma C Wu Q Zhang W Lao G. MicroRNA-23a regulates epithelial-to-mesenchymal transition in endometrial endometrioid adenocarcinoma by targeting SMAD3. Cancer Cell Int (2016) 16(1):67. 10.1186/s12935-016-0342-1 27601936 PMC5011925

[156] Dong PX Ihira K Xiong Y Watari H Hanley SJB Yamada T Reactivation of epigenetically silenced miR-124 reverses the epithelial-to-mesenchymal transition and inhibits invasion in endometrial cancer cells via the direct repression of IQGAP1 expression. Oncotarget (2016) 7(15):20260–70. 10.18632/oncotarget.7754 26934121 PMC4991452

[157] Yoshida S Furukawa N Haruta S Tanase Y Kanayama S Noguchi T Expression profiles of genes involved in poor prognosis of epithelial ovarian carcinoma: a review. Int J Gynecol Cancer (2009) 19(6):992–7. 10.1111/IGC.0b013e3181aaa93a 19820358

[158] Burk U Schubert J Wellner U Schmalhofer O Vincan E Spaderna S A reciprocal repression between ZEB1 and members of the miR-200 family promotes EMT and invasion in cancer cells. EMBO Rep (2008) 9(6):582–9. 10.1038/embor.2008.74 18483486 PMC2396950

[159] Bracken CP Gregory PA Kolesnikoff N Bert AG Wang J Shannon MF A double-negative feedback loop between ZEB1-SIP1 and the miroRNA-200 family regulates epithelial-mesenchymal transition. Cancer Res (2008) 68:7846–54. 10.1158/0008-5472.CAN-08-1942 18829540

[160] Li BL Lu C Lu W Yang TT Qu J Hong X miR-130b is an EMT-related microRNA that targets DICER1 for aggression in endometrial cancer. Med Oncol (2013) 30:484. 10.1007/s12032-013-484-0 23392577

[161] Kinose Y Sawada K Nakamura K Kimura T. The role of microRNAs in ovarian cancer. Biomed Res Int (2014) 2014:249393. 10.1155/2014/249393 25295252 PMC4177088

[162] Jin M Yang Z Ye W Xu H Hua X MicroRNA-150 predicts a favorable prognosis in patients with epitheliala ovarian cancer, and inhibits cell invasion and metastasis by suppressing transcriptional repressor ZEB1. PLoS One (2014) 9:e103965 10.1371/journal.pone.0103965 25090005 PMC4121232

[163] Ge T Yin M Yang M Liu T Lou G MicroRNA-302b suppress human epithelial ovarian cancer cell growth by targeting RUNX1. Cell Physiol Biochem (2014) 34:2209–20. 10.1159/000369664 25562167

[164] Zhang L Li Z Gai F Wang Y MicroRNA-137 supresses tumor growth in epithelial ovarian cancer in vitro and in vivo. Mol Med Rep (2015) 12:3107–14. 10.3892/mmr.2015.3756 25955305

[165] Wang L Mezencev R Ŝvajdler M Benigno BB Mcdonald J Ectopic over-expression of miR-429 induces mesenchymal-to-epithelial transition (MET) and increased drug sensitivity in metastazing ovarian cancer cells. Gynecol Oncol (2014) 134:96–103. 10.1016/j.ygyno.2014.04.055 24802724

[166] Zhou X Hu Y Dai L Wang Y Zhou J Wang W MicroRNA-7 inhibits tumor metastasis and reverses epithelial-mesenchymal transition through AKT/ERK1/2 inactivation by targeting EGFR in epithelial ovarian cancer. PLoS One (2014) 9(5):e96718. 10.1371/journal.pone.0096718 24816687 PMC4016102

[167] Zhou Y Chen Q Qin R Zhang K Li H. MicroRNA-449a reduces cell survival and enhances cisplatin-induced cytotoxicity via downregulation of NOTCH1 in ovarian cancer cells. Tumour Biol (2014) 35(12):12369–78. 10.1007/s13277-014-2551-3 25179844

[168] Parikh A Lee C Joseph P Marchini S Baccarini A Kolev V microRNA-181a has a critical role in ovarian cancer progression through the regulation of the epithelial-mesenchymal transition. Nat Commun (2014) 5:2977. 10.1038/ncomms3977 24394555 PMC3896774

[169] Caldon CE. Estrogen signaling and the DNA damage response in hormone dependent breast cancers. Front Oncol (2014) 4:106. 10.3389/fonc.2014.00106 24860786 PMC4030134

[170] Zhang Z Zhou D Lai Y Liu Y Tao X Wang Q Estrogen induces endometrial cancer cell proliferation and invasion by regulating the fat mass and obesity-associated gene via PI3K/AKT and MAPK signaling pathways. Cancer Lett (2012) 319(1):89–97. 10.1016/j.canlet.2011.12.033 22222214

[171] Mungenast F Thalhammer T. Estrogen biosynthesis and action in ovarian cancer. Front Endocrinol (2014) 5:192. 10.3389/fendo.2014.00192 25429284 PMC4228918

[172] Secky L Svoboda M Klameth L Bajna E Hamilton G Zeillinger R The sulfatase pathway for estrogen formation: targets for the treatment and diagnosis of hormone-associated tumors. J Drug Deliv (2013) 2013:957605. 10.1155/2013/957605 23476785 PMC3586502

[173] Park SH Cheung LWT Wong AST Leung PCK Estrogen regulates snail and slug in the down-regulation of E-cadherin and induces metastatic potential of ovarian cancer cells through estrogen receptor alpha. Mol Endocrinol (2008) 22(9):2085–98. 10.1210/me.2007-0512 18550773 PMC5419456

[174] Lam SS Mak AS Yam JW Cheung AN Ngan HY Wong AS. Targeting estrogen-related receptor alpha inhibits epithelial-to-mesenchymal transition and stem cell properties of ovarian cancer cells. Mol Ther (2014) 22(4):743–51. 10.1038/mt.2014.1 24419103 PMC3982489

[175] Yang YB Zhang JW Zhu YP Zhang ZB Sun H Feng YJ. Follicle-stimulating hormone induced epithelial-mesenchymal transition of epithelial ovarian cancer cells through follicle-stimulating hormone receptor PI3K/Akt-snail signaling pathway. Int J Gynecol Cancer (2014) 24(9):1564–74. 10.1097/Igc.0000000000000279 25340291

[176] Zheng W Lu JJ Luo F Zheng Y Feng Y Felix JC Ovarian epithelial tumor growth promotion by follicle-stimulating hormone and inhibition of the effect by luteinizing hormone. Gynecol Oncol (2000) 76(1):80–8. 10.1006/gyno.1999.5628 10620446

[177] Jeon SY Choi KC Progesterone is a potent substance which inhibits the migration of ovarian cancer cells by reducing epithelial-mesenchymal transition via progesterone receptor-dependent pathway. Endocr Abstr (2015) 37:EP1151 10.1530/endoabs.37.EP1151

[178] Spoelstra NS Manning NG Higashi Y Darling D Singh M Shroyer KR The transcription factor ZEB1 is aberrantly expressed in aggressive uterine cancers. Cancer Res (2006) 66(7):3893–902. 10.1158/0008-5472.Can-05-2881 16585218

[179] Richer JK Jacobsen BM Manning NG Abel MG Wolf DM Horwitz KB Differential gene regulation by the two progesterone receptor isoforms in human breast cancer cells. J Biol Chem (2002) 277(7):5209–18. 10.1074/jbc.M110090200 11717311

[180] Van Der Horst PH Wang Y Vandenput I Kuhne LC Ewing PC Van Ijcken WF Progesterone inhibits epithelial-to-mesenchymal transition in endometrial cancer. PLoS One (2012) 7(1):e30840. 10.1371/journal.pone.0030840 22295114 PMC3266274

[181] Wik E Raeder MB Krakstad C Tovik J Birkeland E Hoivik EA Lack of estrogen receptor-α is associated with epithelial-mesenchymal transition and PI3K alterations in endometrial carcinoma. Clin Cancer Res (2013) 19(5):1094–105. 10.1158/1078-0432.CCR-12-3039 23319822

[182] Zhang H Li H Qi S Liu Z Fu Y Li M Normal endometrial stromal cells regulate 17beta-estradiol-induced epithelial-mesenchymal transition via slug and E-cadherin in endometrial adenocarcinoma cells in vitro. Gynecol Endocrinol (2017) 33(1):82–6. 10.1080/09513590.2016.1203896 27449470

[183] Liu Z Qi S Zhao X Li M Ding S Lu J Metformin inhibits 17beta-estradiol-induced epithelial-to-mesenchymal transition via betaKlotho-related ERK1/2 signaling and AMPKalpha signaling in endometrial adenocarcinoma cells. Oncotarget (2016) 7(16):21315–31. 10.18632/oncotarget.7040 26824324 PMC5008287

[184] Landskron G De La Fuente M Thuwajit P Thuwajit C Hermoso MA. Chronic inflammation and cytokines in the tumor microenvironment. J Immunol Res (2014) 2014:149185. 10.1155/2014/149185 24901008 PMC4036716

[185] Kulbe H Chakravarty P Leinster DA Charles KA Kwong J Thompson RG A dynamic inflammatory cytokine network in the human ovarian cancer microenvironment. Cancer Res (2012) 72(1):66–75. 10.1158/0008-5472.CAN-11-2178 22065722 PMC3252703

[186] Charles KA Kulbe H Soper R Escorcio-Correia M Lawrence T Schultheis A The tumor-promoting actions of TNF-alpha involve TNFR1 and IL-17 in ovarian cancer in mice and humans. J Clin Invest (2009) 119(10):3011–23. 10.1172/JCI39065 19741298 PMC2752076

[187] Kolomeyevskaya N Eng KH Khan AN Grzankowski KS Singel KL Moysich K Cytokine profiling of ascites at primary surgery identifies an interaction of tumor necrosis factor-α and interleukin-6 in predicting reduced progression-free survival in epithelial ovarian cancer. Gynecol Oncol (2015) 138(2):352–7. 10.1016/j.ygyno.2015.04.009 26001328 PMC4522366

[188] Szlosarek PW Grimshaw MJ Kulbe H Wilson JL Wilbanks GD Burke F Expression and regulation of tumor necrosis factor alpha in normal and malignant ovarian epithelium. Mol Cancer Ther (2006) 5(2):382–90. 10.1158/1535-7163.MCT-05-0303 16505113

[189] Wu Y Zhou BP TNF-α/NF-kB/Snail pathway in cancer cell migration and invasion. Br J Cancer (2010) 102(4):639–44. 10.1038/sj.bjc.6605530 20087353 PMC2837572

[190] Li CW Xia W Huo L Lim SO Wu Y Hsu JL Epithelial-mesenchymal transition induced by TNF-α requires NF-kB-mediated transcriptional upregulation of Twist1. Cancer Res (2012) 72(5):1290–300. 10.1158/0008-5472.CAN-11-3123 22253230 PMC3350107

[191] Wang H Wang HS Zhou BH Li CL Zhang F Wang XF Epithelial-mesenchymal transition (EMT) induced by TNF-alpha requires AKT/GSK-3beta-mediated stabilization of snail in colorectal cancer. PLoS One (2013) 8(2):e56664 10.1371/journal.pone.0056664 23431386 PMC3576347

[192] Bates RC Mercurio AM. Tumor necrosis factor-alpha stimulates the epithelial-to-mesenchymal transition of human colonic organoids. Mol Biol Cell (2003) 14(5):1790–800. 10.1091/mbc.E02-09-0583 12802055 PMC165077

[193] Kulbe H Thompson R Wilson JL Robinson S Hagemann T Fatah R The inflammatory cytokine tumor necrosis factor-alpha generates an autocrine tumor-promoting network in epithelial ovarian cancer cells. Cancer Res (2007) 67(2):585–92. 10.1158/0008-5472.CAN-06-2941 17234767 PMC2679985

[194] Jiang YP Wu XH Xing HY Du XY. Role of CXCL12 in metastasis of human ovarian cancer. Chin Med J (Engl) (2007) 120(14):1251–5. 17697577

[195] Jiang YP Wu XH Xing HY Du XY Effect of chemokine CXCL12 and its receptor CXR4 on proliferation, migration and invasion of epithelial ovarian cancer cells. Zhonghua Fu Chan Ke Za Zhi (2007) 42(6):403–7. 17697603

[196] Scotton CJ Wilson JL Scott K Stamp G Wilbanks GD Fricker S Multiple actions of the chemokine CXCL12 on epithelial tumor cells in human ovarian cancer. Cancer Res (2002) 62(20):5930–8. 12384559

[197] Vergara D Merlot B Lucot JP Collinet P Vinatier D Fournier I Epithelial-mesenchymal transition in ovarian cancer. Cancer Lett (2010) 291(1):59–66. 10.1016/j.canlet.2009.09.017 19880243

[198] Lebrun JJ The dual role of TGFbeta in human cancer: from tumor suppression to cancer metastasis. ISRN Mol Biol (2012) 2012:381428 10.5402/2012/381428 27340590 PMC4899619

[199] Higashi T Sasagawa T Inoue M Oka R Shuangying L Saijoh K. Overexpression of latent transforming growth factor-beta 1 (TGF-beta 1) binding protein 1 (LTBP-1) in association with TGF-beta 1 in ovarian carcinoma. Jpn J Cancer Res (2001) 92(5):506–15. 10.1111/j.1349-7006.2001.tb01123.x 11376559 PMC5926747

[200] Yeung TL Leung CS Wong KK Samimi G Thompson MS Liu J TGF-beta modulates ovarian cancer invasion by upregulating CAF-derived versican in the tumor microenvironment. Cancer Res (2013) 73(16):5016–28. 10.1158/0008-5472.CAN-13-0023 23824740 PMC3745588

[201] Zhang Y Tang H Cai J Zhang T Guo J Feng D Ovarian cancer-associated fibroblasts contribute to epithelial ovarian carcinoma metastasis by promoting angiogenesis, lymphanogiogenesis and tumor cell invasion. Cancer Lett (2011) 303(1):47–55. 10.1016/j.canlet.2011.01.011 21310528

[202] Yamamura S Matsumura N Mandai M Huang ZQ Oura T Baba T The activated transforming growth factor-beta signaling pathway in peritoneal metastases is a potential therapeutic target in ovarian cancer. Int J Cancer (2012) 130(1):20–8. 10.1002/ijc.25961 21503873

[203] Basu M Bhattacharya R Ray U Mukhopadhyay S Chatterjee U Roy SS Invasion of ovarian cancer cells is induced by PITX-2-mediated activation of TGF-β and Activin-A. Mol Cancer (2015) 14:162 10.1186/s12943-015-0433-y 26298390 PMC4546816

[204] Qiu X Cheng JC Zhao J Chang HM Leung PC Transforming growth factor-β stimulates human ovarian cancer cell migration by up-regulation connexion 43 expression via Smad2/3 signaling. Cell Signal (2015) 27(10):1956–62. 10.1016/j.cellsig.2015.07.010 26186970

[205] Qiu X Cheng JC Klausen C Chang HM Fan Q Leung PC. EGF-induced connexin43 negatively regulates cell proliferation in human ovarian cancer. J Cell Physiol (2016) 231(1):111–9. 10.1002/jcp.25058 26031779

[206] Cardenas H Vieth E Lee J Segar M Liu Y Nephew KP TGF-beta induces global changes in DNA methylation during the epithelial-to-mesenchymal transition in ovarian cancer cells. Epigenetics (2014) 9(11):1461–72. 10.4161/15592294.2014.971608 25470663 PMC4622747

[207] Mclean K Gong Y Choi Y Deng N Yang K Bai S Human ovarian carcinoma-associated mesenchymal stem cells regulate cancer stem cells and tumorigenesis via altered BMP production. J Clin Invest (2011) 121(8):3206–19. 10.1172/JCI45273 21737876 PMC3148732

[208] Le Page C Puiffe ML Meunier L Zietarska M De Ladurantaye M Tonin PN BMP-2 signaling in ovarian cancer and its association with poor prognosis. J Ovarian Res (2009) 2:4. 10.1186/1757-2215-2-4 19366455 PMC2674440

[209] Thériault BL Shepherd TG Mujoomdar ML Nachtigal MW BMP-4 induces EMT and Rho GTPase activation in human ovarian cancer cells. Carcinogenesis (2007) 28:1153–62. 10.1093/carcin/bgm015 17272306

[210] Quail DF Siegers GM Jewer M Postovit LM Nodal signaling in embryogenesis and tumorigenesis. Int J Biochem Cell Biol (2013) 45(4):885–98. 10.1016/j.biocel.2012.12.021 23291354

[211] Guo Q Ning F Fang R Wang HS Zhang G Quan MY Endogenous Nodal promotes melanoma undergoing epithelial-mesenchymal transition via Snail and Slug in vitro and in vivo. Am J Cancer Res (2015) 5(6):2098–112. 26269769 PMC4529629

[212] Fang R Zhang G Guo Q Ning F Wang H Cai S Nodal promotes aggressive phenotype via Snail-mediated epithelial-mesenchymal transition in murine melanoma. Cancer Lett (2013) 333(1):66–75. 10.1016/j.canlet.2013.01.014 23348697

[213] Quail DF Zhang G Findlay SD Hess DA Postovit LM. Nodal promotes invasive phenotypes via a mitogen-activated protein kinase-dependent pathway. Oncogene (2014) 33(4):461–73. 10.1038/onc.2012.608 23334323 PMC5025281

[214] Xu G Zhong Y Munir S Yang BB Tsang BK Peng C Nodal induces apoptosis and inhibits proliferation in human epithelial ovarian cells via activing receptor-like kinase 7. Clin Endocrinol Metab (2004) 89:5523–34. 10.1210/jc.2004-0893 15531507

[215] Papageorgiou I Nicholls PK Wang F Lackmann M Makanji Y Salamonsen LA Expression of nodal signalling components in cycling human endometrium and in endometrial cancer. Reprod Biol Endocrinol (2009) 7:122. 10.1186/1477-7827-7-122 19874624 PMC2774317

[216] Lindsey S Langhans SA. Crosstalk of oncogenic signaling pathways during epithelial-mesenchymal transition. Front Oncol (2014) 4:358. 10.3389/fonc.2014.00358 25566498 PMC4263086

[217] Colomiere M Findlay J Ackland L Ahmed N Epidermal growth-induced ovarian carcinoma cell migration is associated with JAK2/STAT3 signals and changes in the abundance and localization of alpha6beta1 integrin. Int J Biochem Cell Biol (2009) 41(5):1034–45. 10.1016/j.biocel.2008.09.018 18930836

[218] Colomiere M Ward AC Riley C Trenerry MK Cameron-Smith D Findlay J Cross talk of signals between EGFR and IL-6R through JAK2/STAT3 mediate epithelial-mesenchymal transition in ovarian carcinomas. Br J Cancer (2009) 100(1):134–44. 10.1038/sj.bjc.6604794 19088723 PMC2634691

[219] Matte I Lane D Laplante C Garde-Granger P Rancourt C Piche A. Ovarian cancer ascites enhance the migration of patient-derived peritoneal mesothelial cells via cMet pathway through HGF-dependent and -independent mechanisms. Int J Cancer (2015) 137(2):289–98. 10.1002/ijc.29385 25482018

[220] Nakamura M Ono YJ Kanemura M Tanaka T Hayashi M Terai Y Hepatocyte growth factor secreted by ovarian cancer cells stimulates peritoneal implantation via the mesothelial-mesenchymal transition of the peritoneum. Gynecol Oncol (2015) 139(2):345–54. 10.1016/j.ygyno.2015.08.010 26335595

[221] Rosano L Cianfrocca R Tocci P Spinella F Di Castro V Caprara V Endothelin A receptor/beta-arrestin signaling to the Wnt pathway renders ovarian cancer cells resistant to chemotherapy. Cancer Res (2014) 74(24):7453–64. 10.1158/0008-5472.CAN-13-3133 25377471

[222] Rosano L Spinella F Di Castro V Nicotra MR Dedhar S De Herreros AG Endothelin-1 promotes epithelial-to-mesenchymal transition in human ovarian cancer cells. Cancer Res (2005) 65(24):11649–57. 10.1158/0008-5472.CAN-05-2123 16357176

[223] Rosano L Cianfrocca R Spinella F Di Castro V Nicotra MR Lucidi A Acquisition of chemoresistance and EMT phenotype is linked with activation of the endothelin A receptor pathway in ovarian carcinoma cells. Clin Cancer Res (2011) 17(8):2350–60. 10.1158/1078-0432.Ccr-10-2325 21220476

[224] Muinelo-Romay L Colas E Barbazan J Alonso-Alconada L Alonso-Nocelo M Bouso M High-risk endometrial carcinoma profiling identifies TGF-β1 as a key factor in the initiation of tumor invasion. Mol Cancer Ther (2011) 10(8):1357–66. 10.1158/1535-7163.MCT-10-1019 21613448

[225] Bischof P Campana A. A putative role for oncogenes in trophoblast invasion? Hum Reprod (2000) 15(Suppl 6):51–8. 11261483

[226] Lei X Wang L Yang J Sun LZ. TGFbeta signaling supports survival and metastasis of endometrial cancer cells. Cancer Manag Res (2009) 2009(1):15–24. 20622970 PMC2901109

[227] So KA Min KJ Hong JH Lee JK. Interleukin-6 expression by interactions between gynecologic cancer cells and human mesenchymal stem cells promotes epithelial-mesenchymal transition. Int J Oncol (2015) 47(4):1451–9. 10.3892/ijo.2015.3122 26316317

[228] Planaguma J Abal M Gil-Moreno A Diaz-Fuertes M Monge M Garcia A Up-regulation of ERM/ETV5 correlates with the degree of myometrial infiltration in endometrioid endometrial carcinoma. J Pathol (2005) 207(4):422–9. 10.1002/path.1853 16175655

[229] Colas E Muinelo-Romay L Alonso-Alconada L Llaurado M Monge M Barbazan J ETV5 cooperates with LPP as a sensor of extracellular signals and promotes EMT in endometrial carcinomas. Oncogene (2012) 31(45):4778–88. 10.1038/onc.2011.632 22266854

[230] Pedrola N Devis L Llaurado M Campoy I Martinez-Garcia E Garcia M Nidogen 1 and nuclear protein 1: novel targets of ETV5 transcription factor involved in endometrial cancer invasion. Clin Exp Metastasis (2015) 32(5):467–78. 10.1007/s10585-015-9720-7 25924802

[231] Guo BQ Sallis RE Greenall A Petit MMR Jansen E Young L The LIM domain protein LPP is a coactivator for the ETS domain transcription factor PEA3. Mol Cell Biol (2006) 26(12):4529–38. 10.1128/Mcb.01667-05 16738319 PMC1489114

[232] Beleut M Rajaram RD Caikovski M Ayyanan A Germano D Choi Y Two distinct mechanisms underlie progesterone-induced proliferation in the mammary gland. Proc Natl Acad Sci U S A (2010) 107(7):2989–94. 10.1073/pnas.0915148107 20133621 PMC2840294

[233] Liu Y Wang J Ni T Wang L Wang Y Sun X. CCL20 mediates RANK/RANKL-induced epithelial-mesenchymal transition in endometrial cancer cells. Oncotarget (2016) 7(18):25328–39. 10.18632/oncotarget.8291 27015366 PMC5041907

[234] Li Y Che Q Bian Y Zhou Q Jiang F Tong H Autocrine motility factor promotes epithelial-mesenchymal transition in endometrial cancer via MAPK signaling pathway. Int J Oncol (2015) 47(3):1017–24. 10.3892/ijo.2015.3091 26201353

[235] Quail DF Taylor MJ Postovit LM Microenvironmental regulaton of cancer stem cell phenotype. Curr Stem Cell Res Ther (2012) 7(3):197–216. 10.2174/157488812799859838 22329582

[236] Ke Q Costa M. Hypoxia-inducible factor-1 (HIF-1). Mol Pharmacol (2006) 70(5):1469–80. 10.1124/mol.106.027029 16887934

[237] Höckel M Vaupel P Tumor hypoxia: definitions and current clinical, biological, and molecular aspects. J Natl Cancer Inst (2001) 93(4):266–76. 10.1093/jnci/93.4.266 11181773

[238] Jin Y Wang H Liang X Ma J Wang Y Pathological and prognostic significance of hypoxia-inducible factor 1alpha expression in epithelial ovarian cancer: a meta-analysis. Tumour Biol (2014) 35(8):8149–59. 10.1007/s13277-014-2059-x 24845029

[239] Sahlgren C Gustafsson MV Jin S Poellinger L Lendahl U. Notch signaling mediates hypoxia-induced tumor cell migration and invasion. Proc Natl Acad Sci U S A (2008) 105(17):6392–7. 10.1073/pnas.0802047105 18427106 PMC2359811

[240] Ji F Wang Y Qiu L Li S Zhu J Liang Z Hypoxia inducible factor 1alpha-mediated LOX expression correlates with migration and invasion in epithelial ovarian cancer. Int J Oncol (2013) 42(5):1578–88. 10.3892/ijo.2013.1878 23545606 PMC3661201

[241] Schietke R Warnecke C Wacker I Schödel J Mole DR Campean V The lysyl oxydases LOX and LOX2 are necessary and sufficient to repress E-cadherin in hypoxia: insights into cellular transformation processes mediated by HIF-1. J Biol Chem (2010) 285(9):6658–69. 10.1074/jbc.M109.042424 20026874 PMC2825461

[242] Ding L Zhao L Chen W Liu T Li Z Li X. miR-210, a modulator of hypoxia-induced epithelial-mesenchymal transition in ovarian cancer cell. Int J Clin Exp Med (2015) 8(2):2299–307. 25932166 PMC4402813

[243] Li L Huang K You Y Fu X Hu L Song L Hypoxia-induced miR-210 in epithelial ovarian cancer enhances cancer cell viability via promoting proliferation and inhibiting apoptosis. Int J Oncol (2014) 44(6):2111–20. 10.3892/ijo.2014.2368 24715221

[244] Cheng S Han L Guo J Yang Q Zhou J Yang X. The essential roles of CCR7 in epithelial-to-mesenchymal transition induced by hypoxia in epithelial ovarian carcinomas. Tumour Biol (2014) 35(12):12293–8. 10.1007/s13277-014-2540-6 25168373

[245] Wang Y Ma J Shen H Wang C Sun Y Howell SB Reactive oxygen species promote ovarian cancer progression via the HIF-1alpha/LOX/E-cadherin pathway. Oncol Rep (2014) 32(5):2150–8. 10.3892/or.2014.3448 25174950 PMC4440217

[246] Feng Z Gan H Cai Z Li N Yang Z Lu G Aberrant expression of hypoxia-inducible factor 1α, TWIST and E-cadherin is associated with aggressive tumor phenotypes in endometrioid endometrial carcinoma. Jpn J Clin Oncol (2013) 43(4):396–403. 10.1093/jjco/hys237 23372184

[247] Wang H Bao W Jiang F Che Q Chen Z Wang F Mutant p53 (p53-R248Q) functions as an oncogene in promoting endometrial cancer by up-regulating REGγ. Cancer Lett (2015) 360(2):269–79. 10.1016/j.canlet.2015.02.028 25697482

[248] Asakura T Yamaguchi N Ohkawa K Yoshida K. Proteasome inhibitor-resistant cells cause EMT-induction via suppression of E-cadherin by miR-200 and ZEB1. Int J Oncol (2015) 46(5):2251–60. 10.3892/ijo.2015.2916 25738863

[249] Dong P Karaayvaz M Jia N Kaneuchi M Hamada J Watari H Mutant p53 gain-of-function induces epithelial-mesenchymal transition through modulation of the miR-130b-ZEB1 axis. Oncogene (2013) 32(27):3286–95. 10.1038/onc.2012.334 22847613 PMC3705163

[250] Jiang FZ He YY Wang HH Zhang HL Zhang J Yan XF Mutant p53 induces EZH2 expression and promotes epithelial-mesenchymal transition by disrupting p68-Drosha complex assembly and attenuating miR-26a processing. Oncotarget (2015) 6(42):44660–74. 10.18632/oncotarget.6350 26587974 PMC4792583

[251] Raja FA Chopra N Ledermann JA. Optimal first-line treatment in ovarian cancer. Ann Oncol (2012) 23(Suppl 10):x118–27. 10.1093/annonc/mds315 22987945

[252] Latifi A Abubaker K Castrechini N Ward AC Liongue C Dobill F Cisplatin treatment of primary and metastatic epithelial ovarian carcinomas generates residual cells with mesenchymal stem cell-like profile. J Cell Biochem (2011) 112(10):2850–64. 10.1002/jcb.23199 21618587

[253] Baribeau S Chaudhry P Parent S Asselin E. Resveratrol inhibits cisplatin-induced epithelial-to-mesenchymal transition in ovarian cancer cell lines. PLoS One (2014) 9(1):e86987. 10.1371/journal.pone.0086987 24466305 PMC3899376

[254] Helleman J Jansen MP Burger C Van Der Burg ME Berns EM. Integrated genomics of chemotherapy resistant ovarian cancer: a role for extracellular matrix, TGFbeta and regulating microRNAs. Int J Biochem Cell Biol (2010) 42(1):25–30. 10.1016/j.biocel.2009.10.016 19854294

[255] Ricci F Guffanti F Damia G Ovarian cancer recurrence: role of ovarian stem cells and epithelial-to-mesenchymal transition. J Cancer Sci Ther (2014) 6:298–305. 10.4172/1948-5956.1000284

[256] Obermayr E Castillo-Tong DC Pils D Speiser P Braicu I Van Gorp T Molecular characterization of circulating tumor cells in patients with ovarian cancer improves their prognostic significance – a study of the OVCAD consortium. Gynecol Oncol (2013) 128(1):15–21. 10.1016/j.ygyno.2012.09.021 23017820

[257] Blassl C Kuhlmann JD Webers A Wimberger P Fehm T Neubauer H Gene expression profiling of single circulating tumor cells in ovarian cancer – establishment of a multi-marker gene panel. Mol Oncol (2016) 10(7):1030–42. 10.1016/j.molonc.2016.04.002 27157930 PMC5423187

[258] Alonso-Alconada L Muinelo-Romay L Madissoo K Diaz-Lopez A Krakstad C Trovik J Molecular profiling of circulating tumor cells links plasticity to the metastatic process in endometrial cancer. Mol Cancer (2014) 13:223. 10.1186/1476-4598-13-223 25261936 PMC4190574

[259] Yusuf N Inagaki T Kusunoki S Okabe H Yamada I Matsumoto A SPARC was overexpressed in human endometrial cancer stem-like cells and promoted migration activity. Gynecol Oncol (2014) 134(2):356–63. 10.1016/j.ygyno.2014.04.009 24769035

